# IKZF1 exacerbates the inflammatory response by epigenetically modulating mitochondrial function following acute peritonitis

**DOI:** 10.3389/fimmu.2025.1600903

**Published:** 2025-09-12

**Authors:** Guanya Liu, Pengfei Hu, Ying Dong, Yamin Xu, Zhengyao Yang, Zihao Qi, Yuantao Su

**Affiliations:** ^1^ Department of General Surgery, Huadong Hospital, Fudan University, Shanghai, China; ^2^ Shanghai Key Laboratory of Clinical Geriatric Medicine, Huadong Hospital, Fudan University, Shanghai, China; ^3^ Shanghai Institute of Geriatrics and Gerontology, Huadong Hospital, Fudan University, Shanghai, China; ^4^ Department of Radiology, Huadong Hospital, Fudan University, Shanghai, China; ^5^ Day Care Chemotherapy, Huadong Hospital, Fudan University, Shanghai, China; ^6^ Department of General Pancreatic Surgery, Shanghai General Hospital, Shanghai Jiao Tong University, Shanghai, China

**Keywords:** acute peritonitis, macrophage, mitochondria, inflammation, histone acetylation

## Abstract

**Background:**

Macrophages play pivotal roles in immune homeostasis and host defense against pathogens, yet their excessive activation can lead to tissue damage. Acute peritonitis induced by cecal ligation and puncture (CLP) is associated with dysregulated macrophage-mediated inflammation. IKZF1, a transcription factor, has been implicated in immune regulation, but its role in CLP-induced macrophage activation remains unclear. This study aimed to investigate the molecular mechanism of IKZF1 in regulating inflammatory responses during acute peritonitis.

**Method:**

Using a murine CLP-induced peritonitis model, we analyzed IKZF1 expression in macrophages via RT-qPCR and western blot. Lenalidomide (Len), an IKZF1 inhibitor, was administered to assess its effects on macrophage inflammation and lung injury. Mitochondrial function was evaluated by measuring reactive oxygen species (ROS), ATP levels, and succinate accumulation. Mechanistic studies included chromatin immunoprecipitation (ChIP), co-immunoprecipitation (Co-IP), and HDAC3 activity assays. SDHB expression and acetylation status were analyzed under LPS stimulation, with acetate supplementation used to modulate histone H3K9 acetylation.

**Results:**

IKZF1 expression was significantly upregulated in macrophages during CLP-induced peritonitis. Len treatment suppressed IKZF1, attenuating inflammatory responses and mitigating lung injury. Mechanistically, IKZF1 directly repressed SDHB expression by recruiting HDAC3 to deacetylate SDHB, leading to mitochondrial dysfunction and amplified inflammation. Supplementation with acetate restored H3K9ac levels at the SDHB promoter, counteracting LPS-induced suppression of SDHB. These findings highlight an IKZF1/HDAC3-SDHB-succinate axis driving macrophage hyperactivation.

**Conclusion:**

IKZF1 exacerbates macrophage inflammation in CLP-induced peritonitis by epigenetically silencing SDHB via HDAC3-mediated deacetylation, thereby disrupting mitochondrial metabolism and amplifying pro-inflammatory signals. Targeting IKZF1 or enhancing acetylation may represent novel therapeutic strategies for acute inflammatory conditions. This study establishes IKZF1 as a potential biomarker and therapeutic target for mitigating excessive inflammation in peritonitis.

## Introduction

Peritonitis, characterized by inflammation of the peritoneum, is a life-threatening condition that can result from a variety of causes, including infection, trauma, and iatrogenic factors (1–3). The condition is classified into spontaneous, secondary, and tertiary peritonitis, with secondary peritonitis being the most prevalent, often stemming from intra-abdominal infections or postoperative complications (4, 5). The pathophysiology involves the breach of the gastrointestinal barrier, leading to bacterial translocation and subsequent inflammation (1, 2, 6). Early diagnosis and intervention are crucial due to the high mortality associated with peritonitis, particularly when it progresses to sepsis (5, 7). Despite advancements in management, peritonitis remains a significant challenge in clinical practice, highlighting the need for ongoing research to refine diagnostic and therapeutic approaches.

Macrophages play a pivotal role in the pathogenesis of peritonitis, particularly in initiating and modulating the inflammatory response (2, 8, 9). Previous studies indicate that peritoneal macrophages act as the first line of defense upon infection, releasing a plethora of pro-inflammatory cytokines such as IL-1β and TNF-α to activate the local immune response (10–12). However, an excessive inflammatory response can lead to tissue damage and exacerbate pathological conditions. Overproduction of pro-inflammatory factors not only intensifies the inflammatory reaction in the peritoneum but may also trigger systemic inflammatory response syndrome, leading to organ dysfunction and poor patient prognosis (13). In the course of peritonitis, the hyperactivation of macrophages can lead to a dysregulation of the cytokine network, promoting the infiltration of more leukocytes into the peritoneal cavity and creating a vicious cycle (14). Studies have shown that the activation of peritoneal macrophages in a state of excessive inflammation not only enhances inflammation but also promotes the recruitment of other immune cells by releasing an excess of chemokines such as IL-8 and MCP-1, thereby aggravating the pathological progression of peritonitis (15, 16). Moreover, the role of macrophages in clearing apoptotic cells and tissue repair is also compromised, leading to the persistence of inflammation and delayed tissue repair (17, 18). Therefore, modulating the activity and function of macrophages to prevent their excessive inflammatory response may be a crucial strategy for improving patient outcomes in peritonitis. Exploring the mechanisms by which macrophages function in peritonitis will provide a theoretical foundation for developing new therapeutic approaches.

Ikaros family zinc finger 1 (IKZF1), a zinc finger transcription factor and integral member of the IKAROS gene family, exerts a pivotal influence on lymphocyte development and the regulation of proliferative responses (19). Previous studies have reported that IKZF1 plays a crucial role in coordinating the complex transcriptional program of macrophages in response to pathogen invasion, regulates the epigenetic remodeling of diseased monocytes, and also functions in the proliferation of myeloid precursor cells (19–21). Dysregulation of IKZF1 has been found in the pathology of various diseases, including malignant tumors and autoimmune diseases, suggesting that it is extensively involved in the inflammatory response (22, 23). Given the inflammatory nature of peritonitis, the regulatory function of IKZF1 in the immune response may make it a potential factor in the pathogenesis of peritonitis. Understanding how IKZF1 influences macrophage behavior in such inflammatory settings may provide insights into potential therapeutic strategies for modulating macrophage function in peritonitis and other inflammatory disorders.

In this study, we demonstrated that IKZF1 intensifies inflammatory reactions through the epigenetic regulation of macrophage mitochondria dysfunction in acute peritonitis. This process highlights the role of IKZF1 in shaping the macrophage’s metabolic profile, which in turn influences the intensity and duration of the inflammatory response. The epigenetic changes mediated by IKZF1 can lead to a sustained pro-inflammatory phenotype in macrophages, thereby exacerbating the immune response following acute peritonitis.

## Materials and methods

### Peritonitis animal model

The cecal ligation and puncture (CLP) mouse model of peritonitis was established according to a previously described method (24). In brief, mice were anesthetized via isoflurane inhalation. Subsequently, the cecum was exteriorized through a midline abdominal incision, ligated below the ileocecal valve, and punctured once with a 22-gauge needle to extrude a small amount of feces. The cecum was then replaced into the abdominal cavity, and the abdominal wall was closed in layers. Immediately after surgery, 0.3 mL of saline was administered subcutaneously for fluid resuscitation. For the sham-operated control group, the same surgical procedure was performed without ligation or puncture. Postoperatively, mice were returned to their cages with free access to food and water.

### Peritoneal macrophage isolation

Mice were intraperitoneally injected with 3ml of 3% (w/v) thioglycollate broth to elicit peritoneal macrophage recruitment. After 3 days, 3ml of DMEM was injected into the peritoneal cavity using a 25G needle. The abdomen was gently massaged to facilitate mixing, and then an incision was made from the lower abdomen up to the neck, taking care not to puncture the peritoneum. A sterile cotton-plugged Pasteur pipette was inserted into the peritoneal cavity to collect the peritoneal exudate containing macrophages. The collected cells were centrifuged at 1000 rpm for 5 minutes to pellet the macrophages. The cell pellet was washed and resuspended, and 1×10^6^ cells were seeded into 6-well plates containing RPMI 1640 medium supplemented with 10% heat-inactivated fetal bovine serum (FBS) and 1% Penicillin-Streptomycin antibiotics. After 2 hours, the cells were washed three times with DPBS to remove any non-adherent, non-macrophage cells. The adherent macrophages were then cultured for an additional 2 days at 37°C with 5% CO_2_ to allow for further attachment and stabilization.

### Hematoxylin and eosin staining

Lungs were inflated with 4% paraformaldehyde (PFA) (Beyotime, P0099) and then continuously fixed at 4°C for 24 hours. Subsequently, the lungs were cryoprotected in 30% sucrose and embedded in paraffin. The H&E staining experiment was carried out following the standard H&E protocol. Briefly, the slides were rinsed with water to remove the paraffin. The nuclei were stained with hematoxylin (Beyotime, C0107) for 2 minutes, and the cytoplasm was stained with eosin (Beyotime, C0109) for 3 minutes. After the dehydration and clearing procedures, the slices were mounted with neutral resin.

### Masson’s trichrome staining

Lung tissue sections were initially stained with hematoxylin for 5 minutes. Following this, differentiation was achieved using ethanolic hydrochloric acid (Sigma, 100327), after which the sections were rinsed under running water until they attained a blue-black hue. Subsequently, the sections were subjected to staining with Ponceau 2R (Sigma, P2395) and fuchsin acid (Sigma, F8129) for 10 minutes. After rinsing with distilled water, the sections were differentiated using a 1% phosphomolybdic acid solution (Sigma, P4869) for 2 minutes. The sections were then stained with brilliant green (Sigma, B6756) for 5 minutes and sealed with optical rubber. Examination of the stained sections was conducted under a light microscope, and the extent of fibrosis in the lung tissue was assessed using the Ashcroft scoring method.

### Enzyme−linked immunosorbent assay

For ELISA detection, peritoneal lavage fluid, bronchoalveolar lavage fluid, and cell culture supernatant were collected. The concentrations of TNFα, IL-6, and IL-1β were determined using ELISA kits (BioLegend, LEGEND MAX Mouse IL-6 ELISA Kit, 431307; LEGEND MAX Mouse TNF-α ELISA Kit, 430907; LEGEND MAX Mouse IL-1β ELISA Kit, 432604) following the manufacturer’s protocol.

### Western blots

Cells were lysed in cell lysis buffer (CST, #9803) containing 1 mM PMSF (Beyotime, ST507), protease inhibitor cocktail (1:200) (Millipore, 539134), and incubated for 30min on ice, then centrifuged at 12000g for 15min at 4°C. The cell lysates were heated at 95°C for 10 minutes and separated on 4%-20% SDS-PAGE gradient gels at 120V for 1.5 hours. The membranes were incubated with 5% BSA for 1 hour, followed by rinsing in Tri-buffered saline containing Tween-20 (TBST) (Epizyme, PS103) and incubation overnight at 4°C with primary antibodies anti-IKZF1 (Proteintech, 12016-1-AP), anti-GAPDH (CST, #5174), or anti-SDHB (CST, #92649) in 5% BSA (Sigma, 9048-46-8). Primary antibodies were detected using HRP-conjugated secondary antibodies (CST, #7074). The blots were visualized with the SuperSignal West Pico PLUS (ThermoFisher Scientific, 34580).

### RNA interference

Scrambled RNA control and *Ikzf1*-targeted siRNAs (ThermoFisher Scientific, 162288) were transfected into macrophages in 12-well plates using Lipofectamine RNAiMAX (ThermoFisher Scientific, 13778150) using 5 nM of siRNA per well. After 6 hours, the media were changed to DMEM containing 10% FBS and 1% penicillin-streptomycin, and the cells were incubated for 48 hours.

### Detection of reactive oxygen species

Intracellular ROS levels were quantified using specific fluorescent probes: dihydroethidium (DHE, Thermo Fisher, D11347) for general ROS and mitoSOX Red (Thermo Fisher, M36007) for mitochondrial ROS, following the manufacturer’s protocols. Peritoneal macrophages, post-Len treatment, were stained with either probe for 20 minutes at 37 °C, followed by DMEM washing. Fluorescence imaging was performed using a fluorescence microscope, specifically detecting red fluorescence at an excitation wavelength of 594 nm. Quantitative analysis of fluorescence intensity was conducted using ImageJ software (NIH, USA).

### MDA, 4-HNE, SOD and GSH assays

Cells were lysed in cell lysis buffer (CST, #9803) containing 1 mM PMSF (Beyotime, ST507), protease inhibitor cocktail (1:200) (Millipore, 539134) and incubated for 30min on ice, then centrifuged at 4°C at 12000g for 15min. The obtained supernatants were used to determine MDA (Beyotime, S0131S), 4-HNE (Sangon Biotech, D751041), SOD (Beyotime, S0101S), and GSH (Sangon Biotech, D751008) with commercially available kits according to the manufacturer’s instructions.

### Measurement of mitochondrial membrane potential

Mitochondrial membrane potential was assessed using the JC-1 fluorescent probe (Enhanced Mitochondrial Membrane Potential Assay Kit, Beyotime, C2003S). Following the experimental protocol, cells were incubated with 100 μL of freshly prepared JC-1 working solution at 37°C in a 5% CO_2_ atmosphere for 20 minutes. Post-incubation, cells were subjected to centrifugation at 500 × g for 5 minutes, followed by two PBS washes to eliminate excess dye. ΔΨm was evaluated through fluorescence microscopy, with JC-1 exhibiting red fluorescence (aggregates) in polarized mitochondria and green fluorescence (monomers) in depolarized mitochondria.

### Seahorse analysis

Mitochondrial stress tests in macrophages were evaluated using the Seahorse XF96 Extracellular Flux Analyzer (Seahorse Bioscience). Cells were seeded at 2×10^4^ cells/well in XF96 microplates and analyzed in XF DMEM assay medium (pH 7.4, Seahorse Bioscience, 103575-100) according to standard protocols. The mitochondrial stress assay was performed using sequential injections of metabolic modulators: 2 µM oligomycin (ATP synthase inhibitor), 1 µM carbonyl cyanide-4-(trifluoromethoxy) phenylhydrazone (FCCP, uncoupler), and 2 µM rotenone/antimycin A (complex I/III inhibitors). Following three baseline measurements, compounds were automatically delivered through the instrument’s injection ports. Real-time oxygen consumption rate (OCR) was recorded at 7–8-minute intervals using Wave 2.6 software (Agilent Technologies).

### Mitochondrial complex I–V activity assay

The mitochondria from mouse macrophages were isolated and their activities were assessed using the CheKine™ Micro Mitochondrial Complex Activity Assay Kit (Abbkine, Wuhan, China), following the protocol provided by the manufacturer.

### Plasmid construction and transfection

The expression plasmids encoding flag-tagged HDAC3 were constructed by PCR cloning into the pcDNA3.1 eukaryotic expression vector. All constructs were confirmed by DNA sequencing. Plasmids were transiently transfected into peritoneal macrophages using Advanced DNA RNA Transfection Reagent (ZETA life, AD600150).

### Real-time quantitative real-time PCR and RT-PCR

Total RNA was extracted from macrophages using the Super FastPure Cell RNA Isolation Kit (Vazyme, RC102-01). The quality and concentration of RNA were determined by absorbance at 260 and 280nm using a NanoDrop spectrophotometer. Complementary DNA was synthesized from 0.5-1 μg of RNA, which had a 260/280 ratio of >1.8, using HiScript III All-in-one RT SuperMix Perfect for qPCR Kit (Vazyme, R333-01). Quantitative real-time PCR assays were carried out in technical triplicate on a Quant Studio 7 Real-Time PCR System (Applied Biosystems) with the utilization of PowerUp SYBR Green (Thermofisher, A25742) and a primer concentration set at 5 nM. The sequences of the primers are presented in **Supplementary Table S1**. *Gapdh* was selected as the endogenous reference gene for the gene expression analysis. The fold change in enrichment, normalized to the control samples, was computed using the 2^−ΔΔC^.

### Immunofluorescence staining

Immunofluorescence staining was performed on macrophage cultures grown in glass-bottom dishes. After PBS washing, cells were fixed with 4% paraformaldehyde and blocked with 5% bovine serum albumin (BSA) for 1h at room temperature. Primary antibodies against IKZF1 (Proteintech, 12016-1-AP) and F4/80 (Abcam, ab6640) were applied and incubated at 4°C overnight. Following PBS washes, cells were treated with fluorophore-conjugated secondary antibodies for 1h at room temperature. Nuclei were counterstained using ProLong Gold Antifade Mountant containing DAPI (Thermofisher, P36931). Fluorescent images were acquired using a Leica DMI6000B epifluorescence microscope with LAS AF imaging software (Leica Microsystems), and quantitative analysis was performed using ImageJ (NIH, USA).

### Flow cytometry

For flow cytometric analysis, cells were collected on ice in a solution of 10 mM EDTA in PBS and subsequently fixed with 4% paraformaldehyde. Cells were incubated with 1:100 TruStain FcX (anti-mouse CD16/32) antibody (Biolegend, 101301) in Pharmingen Stain Buffer (BSA) (BD Biosciences, 554656) for 30 minutes on ice to block non-specific binding of immunoglobulin to the Fc receptors, followed by incubation for 1 hour on ice with 1:50 PE anti-mouse CD11b (Biolegend, 101207), APC anti-mouse Ly-6G (Biolegend, 127613), and BV650 anti-mouse CD45 (Biolegend, 103151). Cells were rinsed twice and then resuspended in fresh buffer for analysis on a BD FACSCanto II flow cytometer using BD FACSDiva software (BD Biosciences). To detect apoptosis, cells were washed twice and then resuspended in annexin V binding buffer supplemented with a 1:20 dilution of FITC annexin V solution (Biolegend, 640906). Following a 15-minute incubation, the cells were subjected to flow cytometric analysis using the previously described flow cytometer. The resulting data were processed and analyzed with FlowJo 10.8.1 software (BD Biosciences).

### Chromatin immunoprecipitation

Isolated macrophages were subjected to crosslinking with 1% formaldehyde, followed by cell lysis. The nuclear fraction was then sonicated using an S220 Focused-ultrasonicator (Covaris) to fragment the DNA. Subsequent steps were performed using the Chromatin Immunoprecipitation (ChIP) Assay Kit (Byeotime, P2078) in accordance with the manufacturer’s instructions. For the ChIP assays, 5 μg of specific antibodies, including anti-IKZF1, anti-HDAC3 (CST, #85057), and H3K9ac antibody (CST, #9649), or an equivalent amount of goat IgG (CST, #2729), was utilized. DNA was extracted from the antibody–protein–DNA complexes via phenol/chloroform extraction and ethanol precipitation. RT-qPCR was subsequently conducted using the primer sets detailed in **Supplementary Table S2**. Normal rabbit IgG served as the negative control antibody.

### Immunoprecipitation

Cell lysates were prepared using a cell lysis buffer, and the protein concentration was quantified via the BCA method with the Pierce BCA Protein Assay Kit (ThermoFisher, A55864). A total of 300 µg of protein was aliquoted for immunoprecipitation. For each sample, 1 µg of the specific antibody was added and incubated overnight at 4°C. Subsequently, 20 µl of Protein A/G Beads (MedChemExpress, HY-K0202) was introduced and incubated at 4°C for an additional 2 hours to capture the immunocomplexes. These complexes were then subjected to five washes with IP lysis buffer, followed by resuspension in SDS buffer (Beyotime, P0013G) in preparation for Western blot analysis.

### Statistical analysis

Statistical analyses were conducted using GraphPad Prism 10.0 (GraphPad Software, CA, USA). Continuous variables were expressed as mean ± standard deviation (SD). Intergroup comparisons were assessed using a two-tailed Student’s t-test for two-group analyses, while one-way analysis of variance (ANOVA) was employed for multiple group comparisons. A p-value threshold of <0.05 was established to determine statistical significance.

## Results

### IKZF1 expression is upregulated in macrophages in CLP-induced acute peritonitis mice

**Figure 1:**
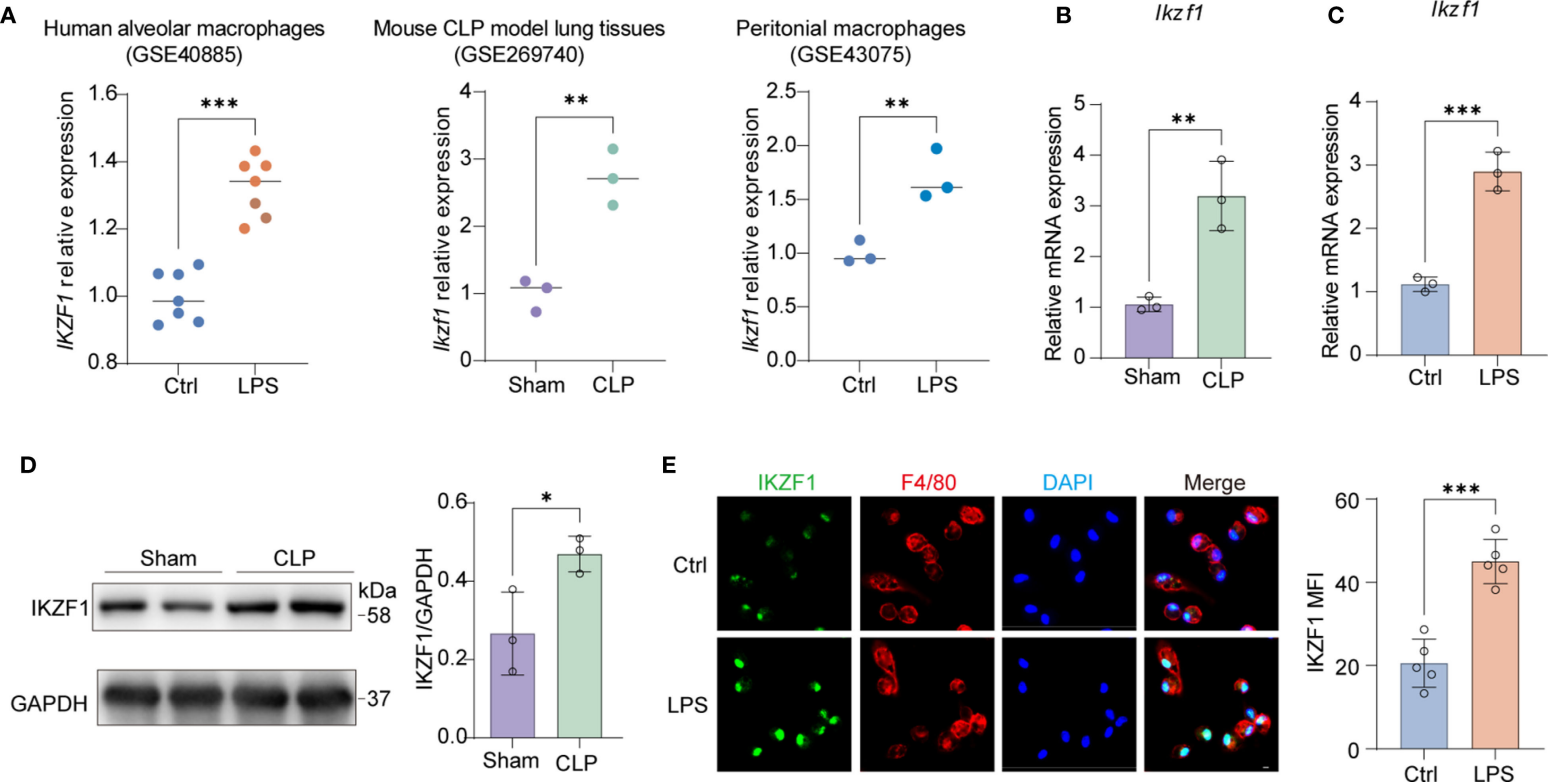
The upregulation of macrophage IKZF1 in CLP-induced peritonitis. **(A)** The expression level of IKZF1 of human alveolar macrophages induced by LPS (GSE40885, Ctrl = 7; LPS=7), mouse lung tissue induced by cecal ligation and puncture (CLP) (GSE269740, Sham = 3; CLP=3), and mouse peritoneal macrophages induced by LPS (GSE43075, Ctrl = 3; LPS=3) from public datasets. **(B)** RT-qPCR analysis of *Ikzf1* in macrophages from the CLP-induced sepsis mice model or sham surgery. (n=3 per group). **(C)** RT-qPCR analysis of *Ikzf1* in macrophages treated with LPS or PBS (Ctrl). (n=3 per group). **(D)** Western blot analysis of IKZF1 in macrophages from the CLP-induced mice sepsis model or sham surgery. (n=3 per group). **(E)** Immunofluorescence analysis of IKZF1 in macrophages treated with LPS or PBS (Ctrl). (n =5 per group, scale bar = 40 μm). All data are represented as mean ± SD. Student’s t-test **(A–E)**. *p < 0.05, **p < 0.01; ***p < 0.001.

To elucidate the regulatory mechanisms involved in peritonitis, we conducted an extensive search of public databases to identify potential inflammatory modulators. Our investigation revealed a notable upregulation of the transcription factor IKZF1 across various infection-associated inflammatory conditions. Cross-dataset evaluation (GSE40885, GSE269740, GSE43075) revealed consistent upregulation of this transcription factor in multiple infection-associated inflammatory contexts, particularly in LPS-challenged alveolar macrophages (25), CLP-induced lung injury models (26), and LPS-stimulated peritoneal macrophages (27) (**Figure 1A**). This conserved expression pattern across diverse infection models suggested a fundamental role for IKZF1 in macrophage-mediated inflammatory responses. To validate these findings, we established a CLP-induced acute peritonitis model demonstrating significant elevation of *Ikzf1* transcript levels in peritoneal macrophages compared to controls (**Figure 1B**). Complementary *in vitro* experiments showed that LPS stimulation induced rapid *Ikzf1* mRNA upregulation in primary peritoneal macrophages within 6 hours (**Figure 1C**). Western blot analysis confirmed corresponding increases in IKZF1 protein expression in peritoneal macrophages isolated from CLP-challenged mice (**Figure 1D**). Immunofluorescence quantification further reinforced these findings, revealing a 2.2-fold increase in nuclear IKZF1 fluorescence intensity following LPS treatment (**Figure 1E**). Collectively, these findings suggest that the elevated IKZF1 expression in peritoneal macrophages may play a pivotal role in the pathogenesis of acute peritonitis.

### IKZF1 inhibition ameliorates CLP and LPS-induced macrophage inflammatory responses

**Figure 2:**
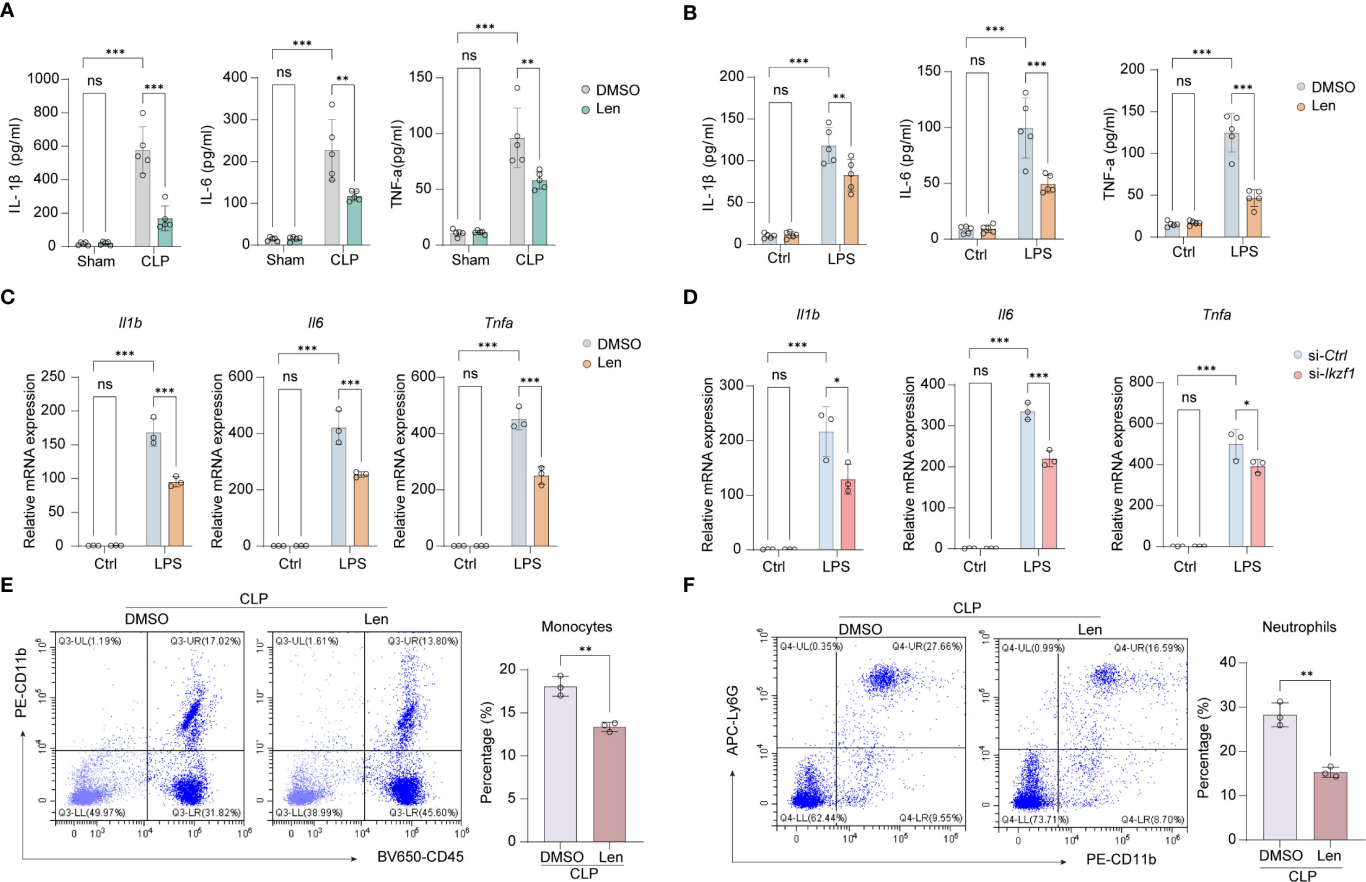
Inhibitor of IKZF1 reduces CLP-induced inflammatory responses. **(A)** ELISA analysis of peritoneal lavage fluid cytokines (IL-1β, IL-6, and TNF-α) from mice treated with the IKZF1 inhibitor Len or DMSO after CLP or sham surgery. (n=5 per group). **(B)** ELISA analysis of cytokines (IL-1β, IL-6, and TNF-α) in culture supernatants of peritoneal macrophages isolated from mice treated with the IKZF1 inhibitor Len or DMSO after CLP or sham operation. (n=5 per group). **(C)** RT-qPCR analysis of *Il1b*, *Il6* and *Tnfα* in peritoneal macrophages treated with Len or DMSO following LPS (100 ng/ml) stimulation for 4h or left untreated (Ctrl). (n=3 per group). **(D)** RT-qPCR analysis of *Il1b*, *Il6*, and *Tnfα* in peritoneal macrophage cells transfected with control siRNA (siCtrl) or *Ikzf1* siRNA (siI*kzf1*) for 48 hours followed by LPS (100 ng/ml) stimulation for 4h or left untreated (Ctrl). (n=3 per group). **(E, F)** Flow cytometry analysis of the number of monocytes and neutrophils in peritoneal lavage fluid after CLP-induced mice. (n=3 per group). All data are represented as mean ± SD. Student’s t-test **(E, F)**; Two-way ANOVA **(A–D)**. *p < 0.05, **p < 0.01; ***p < 0.001.

Len primarily targets cereblon, which is a substrate receptor component of the CRL4^CRBN^ E3 ubiquitin ligase complex (28). This E3 ubiquitin ligase is composed of damaged DNA-binding protein 1, cullin 4a, and regulator of cullins 1. Len modulates the substrate specificity of the CRL4^CRBN^ E3 ubiquitin ligase, selectively promoting the ubiquitination and degradation of the IKZF1 protein. To ascertain the potential role of Len in mitigating acute peritonitis by inhibiting IKZF1, we conducted a series of experiments. Initially, we measured inflammatory cytokines in peritoneal lavage fluid and discovered that Len markedly reduced the CLP-induced peritoneal inflammatory response (**Figure 2A**, **Supplementary Figure 1A**). Following the establishment of the CLP model, we observed a significant reduction in immune cell infiltration in the peritoneal tissue after treatment with Len (**Supplementary Figure 1B**). Furthermore, in cultured peritoneal macrophages, we noted a substantial reduction in protein and mRNA expression of inflammatory cytokines in LPS-stimulated macrophages after Len treatment (**Figures 2B, C**, **Supplementary Figure 1C**). In addition, we also observed that the mRNA expression of the anti-inflammatory cytokine *Il10* significantly increased after IKZF1 inhibition (**Supplementary Figure 1D**). We further evaluated the expression of markers of alternatively activated macrophages and found that inhibition of IKZF1 could significantly promote the expression of *Arg1* and *Mrc1*(**Supplementary Figure 1E**). The flow cytometry results further demonstrated that the IKZF1 inhibitor effectively promotes the polarization of macrophages toward an alternatively activated phenotype in peritoneal macrophages from CLP-treated mice (**Supplementary Figure 1F**). To further confirm the regulatory effect of IKZF1 on macrophage inflammation, we designed and synthesized siRNA specifically targeting *Ikzf1* mRNA (**Supplementary Figure 1G**). The results showed that treatment with *Ikzf1* siRNA significantly inhibited the inflammatory response of macrophages induced by LPS (**Figure 2D**). Subsequently, we analyzed the effect of Len on the chemotaxis of immune cells. In peritoneal macrophages isolated from CLP-treated mice, the expression of chemokines *Cxcl1* and *Cxcl2* was significantly suppressed by Len treatment (**Supplementary Figure 1H**). Furthermore, the levels of CXCL1 and CXCL2 in the peritoneal lavage fluid of these mice were also markedly decreased (**Supplementary Figure 1I**). Assisted by flow cytometry, we found that Len markedly suppressed the proportion of peripheral blood monocytes and neutrophils stimulated by CLP (**Figures 2E, F**, **Supplementary Figure 1J**). Collectively, these findings indicate that the suppression of *Ikzf1* expression leads to a notable amelioration of the inflammatory response in acute peritonitis, suggesting that IKZF1 could serve as a promising therapeutic target for the condition.

### IKZF1 inhibition ameliorates CLP-induced pulmonary injury

**Figure 3:**
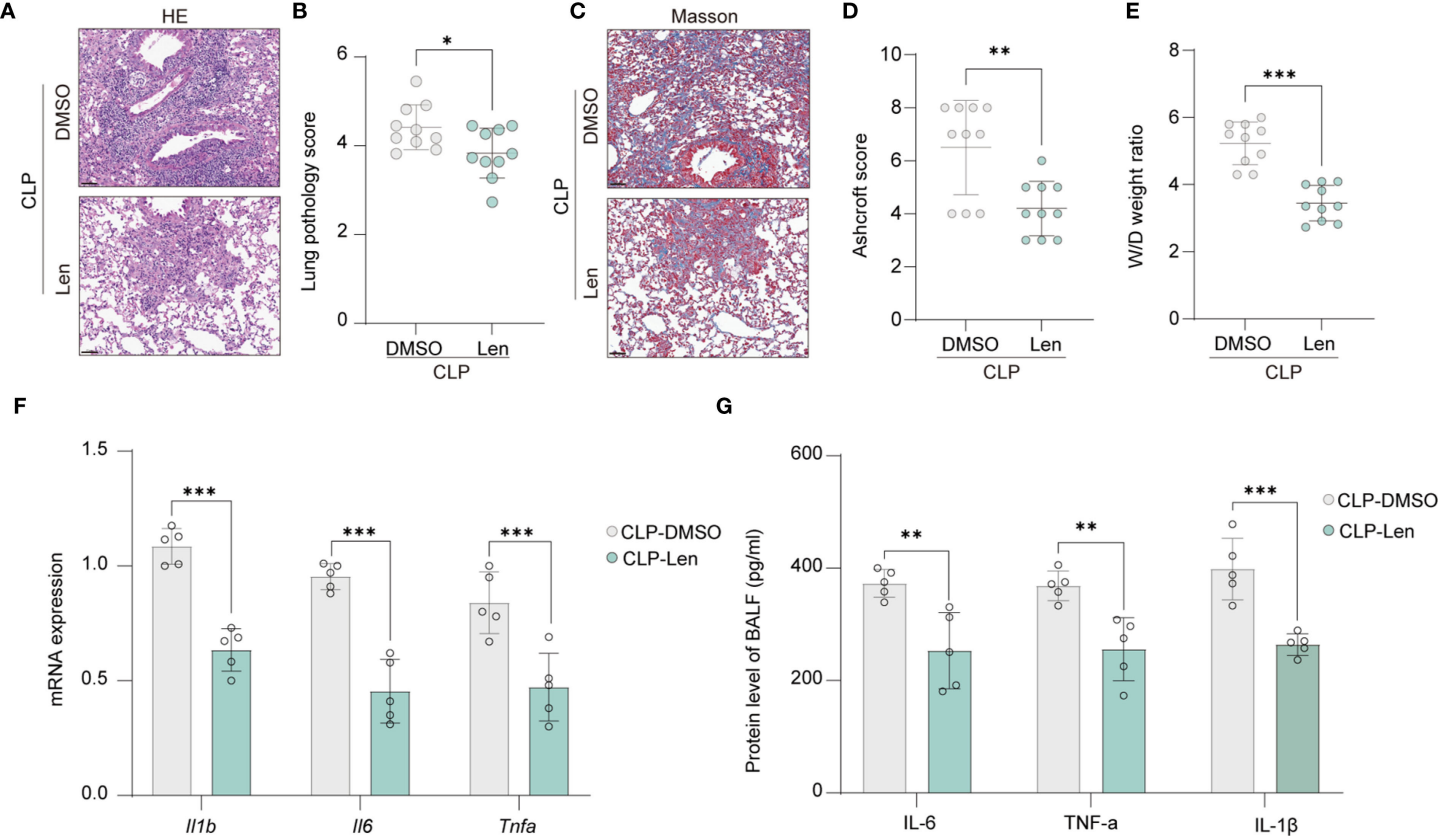
Inhibition of IKZF1 improves CLP-induced pulmonary damage. **(A)** H&E staining of lung tissue histological sections from mice treated with the IKZF1 inhibitor Len or DMSO as a control after CLP-induced injury. **(B)** Smith score of lung tissue pathology injury from mice as in A. (n=10 per group). **(C)** Masson staining of lung tissues histological sections from mice as in A. **(D)** Ashcroft score of lung tissue fibrosis from mice as in A. (n=10 per group). **(E)** Wet weight to Dry weight ratio of lung tissues from mice as in A. (n=10 per group). **(F, G)** RT-qPCR analysis and ELISA of *Il1β*, *Il6*, and *Tnfα* in lung tissues and bronchoalveolar lavage fluid (BALF) from mice as in A. (n=5 per group). All data are represented as mean ± SD. Student’s t-test **(B, D–G)**. *p < 0.05, **p < 0.01; ***p < 0.001.

Acute peritonitis is an inflammatory condition of the peritoneum, often caused by infection or injury, which can lead to systemic inflammatory response syndrome and multiple organ dysfunction. One of the complications that may arise from acute peritonitis is pulmonary injury. We are further evaluating the impact of IKZF1 inhibition on lung injury triggered by acute peritonitis. Through HE staining, we observed a significant reduction in immune cell infiltration in lung tissue following IKZF1 inhibition (**Figure 3A**). The pathological score of lung tissue also significantly decreased after IKZF1 inhibition (**Figure 3B**). Following lung injury, immune cells are pivotal in orchestrating the inflammatory response by secreting a spectrum of inflammatory mediators. These mediators are instrumental in the recruitment and activation of fibroblasts, which are crucial for the progression of fibrosis. Masson’s Trichrome staining showed a significant reduction in fibrosis in lung tissue treated with Len (**Figure 3C**), and the fibrosis score also significantly decreased after IKZF1 inhibition (**Figure 3D**). In addition, the expression of fibrosis-related genes and the collagen content in lung tissue were also inhibited by IKZF1 inhibition (**Supplementary Figures 2A, B**). Subsequently, we collected lung tissue and assessed the wet/dry (W/D) weight ratio of the injured lung tissue, indicating that lung injury significantly decreased after IKZF1 inhibition (**Figure 3E**). We further detected the expression of inflammatory cytokines in injured lung tissue, and the results showed that Len significantly inhibited the expression of pulmonary inflammatory cytokines caused by acute peritonitis (**Figure 3F**). Additionally, in the bronchoalveolar lavage fluid, we also found that the production of inflammatory cytokines significantly decreased after IKZF1 inhibition (**Figure 3G**). These findings indicate that the IKZF1 inhibitor, Len, can substantially mitigate lung injury triggered by acute peritonitis, suggesting that IKZF1 inhibition may notably decrease the incidence of complications associated with acute peritonitis.

### IKZF1 inhibition suppresses macrophage mitochondrial dysfunction

**Figure 4:**
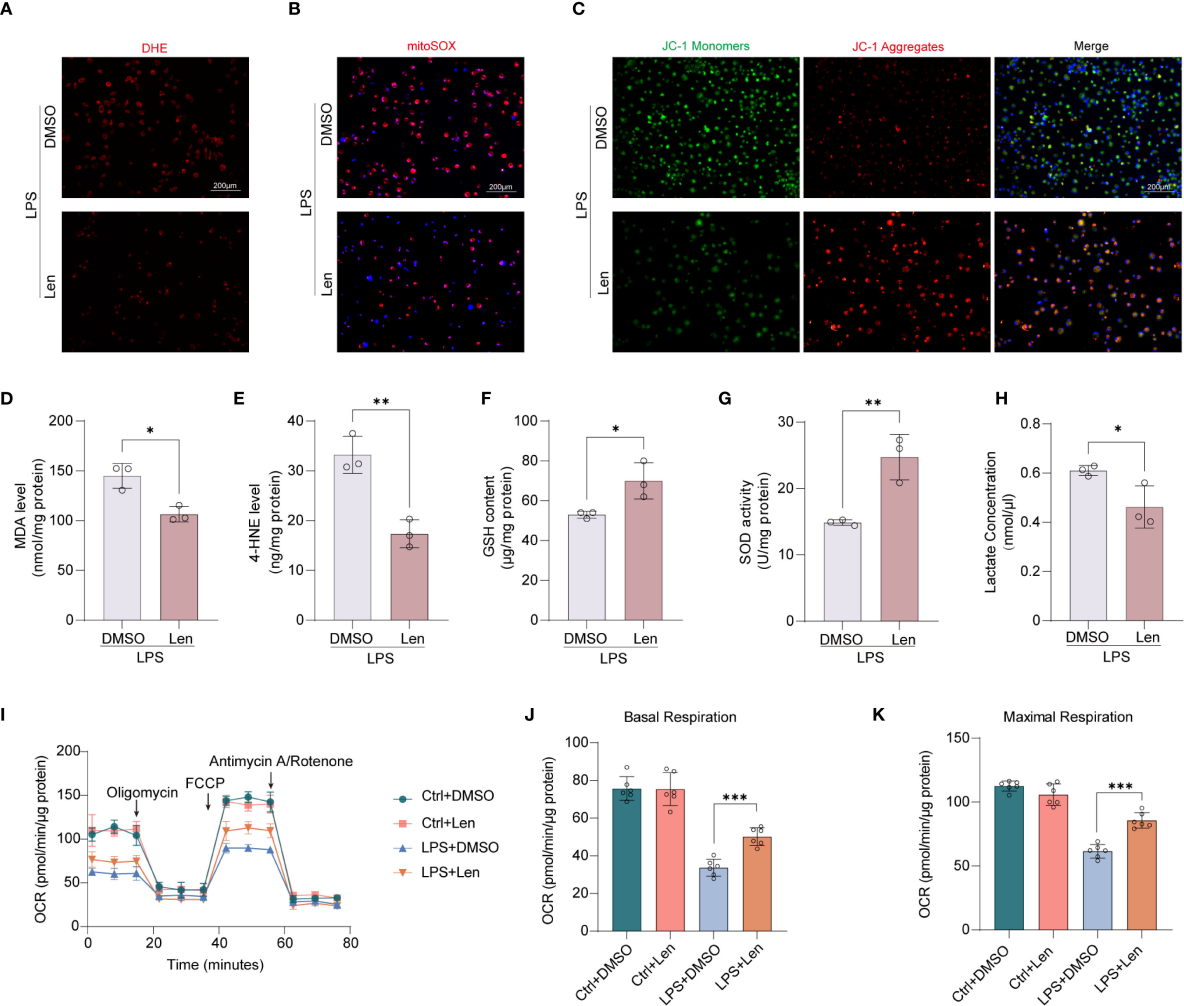
Inhibition of IKZF1 restores mitochondrial function in peritoneal macrophages. **(A)** Representative image of the intracellular ROS levels in peritoneal macrophages subjected to LPS (100 ng/ml) stimulation for 4h or left untreated (Ctrl), followed by treatment with the IKZF1 inhibitor Len or DMSO, and then stained with DHE. (Scale bar: 200 µm). **(B)** Representative images of peritoneal macrophages stained with MitoSOX subjected to LPS (100 ng/ml) stimulation for 4h or left untreated (Ctrl), followed by treatment with the IKZF1 inhibitor Len or DMSO. (Scale bar: 200µm). **(C)** Representative images of JC-1 fluorescence in peritoneal macrophages as in B. Red fluorescence represents the mitochondrial aggregate JC-1 and green fluorescence indicates the monomeric JC-1. (Scale bar: 200µm). **(D–H)** The levels of MDA, 4-HEN, GSH, SOD, and Lactate in peritoneal macrophage as in B. (n=3 per group). **(I)** Oxygen consumption rate (OCR) analysis of peritoneal macrophages as in B. **(J, K)** Relative basal respiration levels analysis and maximal respiration levels analysis of peritoneal macrophages as in B. (n=6 per group). All data are represented as mean ± SD. Student’s t-test **(D–H, J, K)**. *p < 0.05; **p < 0.01; ***p < 0.001.

Mitochondrial dysfunction is increasingly recognized as a critical factor in the development and perpetuation of macrophage inflammation. We further explored whether IKZF1 inhibition affects macrophage mitochondrial function. Using DHE staining analysis, we observed that the intracellular levels of reactive oxygen species (ROS) were significantly reduced following IKZF1 inhibition (**Figure 4A**, **Supplementary Figure 3A**). The ROS release in the peritoneal lavage was also significantly reduced after IKZF1 inhibition (**Supplementary Figure 3B**). We further used the mitochondrial-specific probe mitoSOX to detect the level of ROS derived from mitochondria. The results showed that Len significantly inhibited the generation of mitochondrial ROS (**Figure 4B**, **Supplementary Figure 3C**). JC-1 was employed to assess changes in mitochondrial membrane potential, and as expected, there was a decrease in JC-1 monomers and an increase in JC-1 aggregates in macrophages in the presence of Len, suggesting that Len attenuates LPS-induced mitochondrial dysfunction (**Figure 4C**, **Supplementary Figure 3D**). To investigate the impact of Len on oxidative stress, we measured the levels of MDA, 4-HNE, SOD, and GSH in LPS-stimulated macrophages. We found that treatment with Len significantly reduced the content of oxidative stress markers MDA and 4-HNE (**Figures 4D, E**, **Supplementary Figure 3E**), while the levels of antioxidant molecules SOD and GSH were significantly increased (**Figures 4F, G**, **Supplementary Figure 3F**). This suggests that the IKZF1 inhibitor Len can effectively suppress LPS-induced oxidative stress responses in macrophages. LPS triggers a metabolic shift in macrophages towards glycolysis, which in turn leads to the substantial generation of lactate. We subsequently evaluated the effect of Len on lactate production within macrophages and discovered that Len markedly suppressed the LPS-induced increase in lactate production (**Figure 4H**, **Supplementary Figure 3G**). The function of mitochondria is closely related to their morphology. We subsequently assessed whether IKZF1 inhibition affects mitochondrial fusion and fission. Through detection, we found that IKZF1 does not affect the expression of regulatory molecules related to mitochondrial fusion and fission (**Supplementary Figures 3H, I**). We delved deeper into the modulatory role of IKZF1 in mitochondrial function, particularly during inflammatory responses. Our results indicate that the IKZF1 inhibitor Len significantly improved mitochondrial dysfunction caused by LPS, as evidenced by a clear change in the rate of oxygen consumption (**Figure 4I**). Further analysis revealed that both basal and maximal respiratory capacities of peritoneal macrophages treated with Len were significantly increased (**Figures 4J, K**). In addition, the ATP-linked respiratory but not proton leak also significantly increased after IKZF1 inhibition (**Supplementary Figures 3J, K**). Therefore, our evidence suggests that inhibition of IKZF1 by Len significantly ameliorates mitochondrial dysfunction in peritoneal macrophages.

### IKZF1 directly inhibits the expression of SDHB

**Figure 5:**
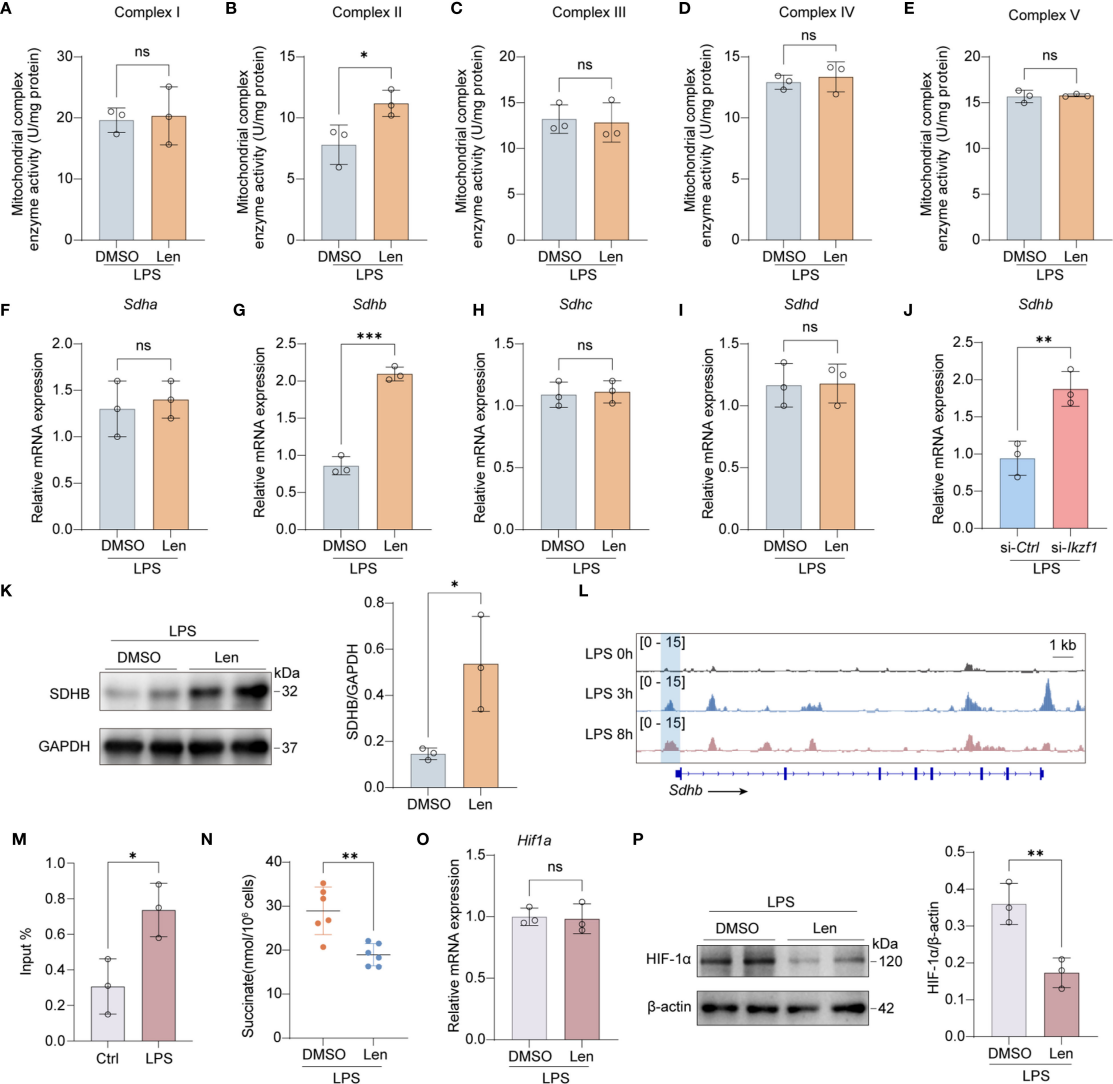
Inhibition of IKZF1 promotes SDHB expression. **(A–E)** Mitochondrial electron transport complex I-V activity in peritoneal macrophages treated with the IKZF1 inhibitor Len or DMSO followed by LPS (100 ng/ml) stimulation for 4h. (n=3 per group). **(F–I)** RT-qPCR analysis of the indicated genes in peritoneal macrophages treated with the IKZF1 inhibitor Len or DMSO followed by LPS (100 ng/ml) stimulation for 4h. (n=3 per group). **(J)** RT-qPCR analysis of *Shdb* in peritoneal macrophages transfected with control siRNA (siCtrl) or *Ikzf1* siRNA (siI*kzf1*) for 48 hours followed by LPS (100 ng/ml) stimulation for 4h. (n=3 per group). **(K)** Western blot analysis of SHDB in peritoneal macrophages treated with IKZF1 inhibitor Len or DMSO, followed by LPS (100 ng/ml) stimulation for 4h. (n=3 per group). **(L)** IGV analysis of IKZF1 enrichment at *Shdb* promoter in peritoneal macrophages at the indicated time for LPS stimulation from public datasets (GSE93599). **(M)** ChIP-qPCR analysis of IKZF1 enrichment at *Shdb* promoter in peritoneal macrophages treated with LPS (100 ng/ml) for 4h. (n=3 per group). **(N)** Evaluation of succinate level in LPS-simulation peritoneal macrophages. (n=6 per group). **(O)** RT-qPCR analysis of *Hif1a* genes in peritoneal macrophages treated with the IKZF1 inhibitor Len or DMSO followed by LPS (100 ng/ml) stimulation for 4h. (n=3 per group). **(P)** Western blot analysis of HIF-1α in peritoneal macrophages treated with IKZF1 inhibitor Len or DMSO, followed by LPS (100 ng/ml) stimulation for 4h (n=3 per group). All data are represented as mean ± SD. Student’s t-test **(A–J, M, N)**. *p < 0.05; **p < 0.01; ***p < 0.001; ns, non-significant.

The respiratory chain is integral to mitochondrial function, influencing immune responses, ROS generation, and energy production, all critical during inflammation and various disease states. We subsequently investigated the potential role of IKZF1 in modulating the mitochondrial respiratory chain, a crucial component of cellular respiration and energy metabolism. We initially assessed the activity of the mitochondrial respiratory chain. The results indicated that the inhibition of IKZF1 significantly improved the activity of mitochondrial respiratory chain complex II, while there were no significant differences in complexes I, III, IV, and V (**Figures 5A–E**), suggesting that IKZF1 might affect the expression of succinate dehydrogenase. We further analyzed the expression of the four family members of succinate dehydrogenase and found that the inhibition of IKZF1 significantly promoted the expression of *Sdhb* (**Figures 5F–I**). Using siRNA targeting *Ikzf1*, we further confirmed the suppressive effect of IKZF1 on *Sdhb* expression (**Figure 5J**). Similarly, Len also significantly increased the protein levels of SDHB (**Figure 5K**). To elucidate the mechanism by which IKZF1 regulates SDHB expression in macrophages after LPS stimulation, we analyzed the potential binding sites identified between IKZF1 and *Sdhb* in peritoneal macrophages after LPS stimulation. According to ChIP-Seq analyses from previous studies (GSE93599), the binding of IKZF1 to the promoter region of Sdhb gradually increases with prolonged stimulation (**Figure 5L**). Our subsequent ChIP-qPCR analyses confirmed that IKZF1 enrichment at the *Sdhb* promoter region was markedly increased following LPS stimulation (**Figure 5M**). SDHB catalyzes the conversion of succinate to fumarate, and our detection showed that Len significantly reduced the accumulation of succinate in macrophages (**Figure 5N**). Previous studies have shown that succinate accumulation can prevent the degradation of HIF-1α, thereby exacerbating the inflammatory response of macrophages. We found that IKZF1 inhibition significantly promotes the degradation of HIF-1α without affecting its transcriptional level (**Figures 5O, P**), further confirming that IKZF1 exacerbates CLP-induced peritonitis by promoting succinate accumulation. These results indicate that within an inflammatory milieu, IKZF1 is capable of binding to and repressing *Sdhb* expression, leading to the accumulation of pro-inflammatory factors such as succinate and ROS, which in turn exacerbate the inflammatory response of macrophages. Conversely, the suppression of IKZF1 significantly mitigates the inflammatory response in macrophages by restoring *Sdhb* expression.

### IKZF1 engages HDAC3 to facilitate SDHB deacetylation

**Figure 6:**
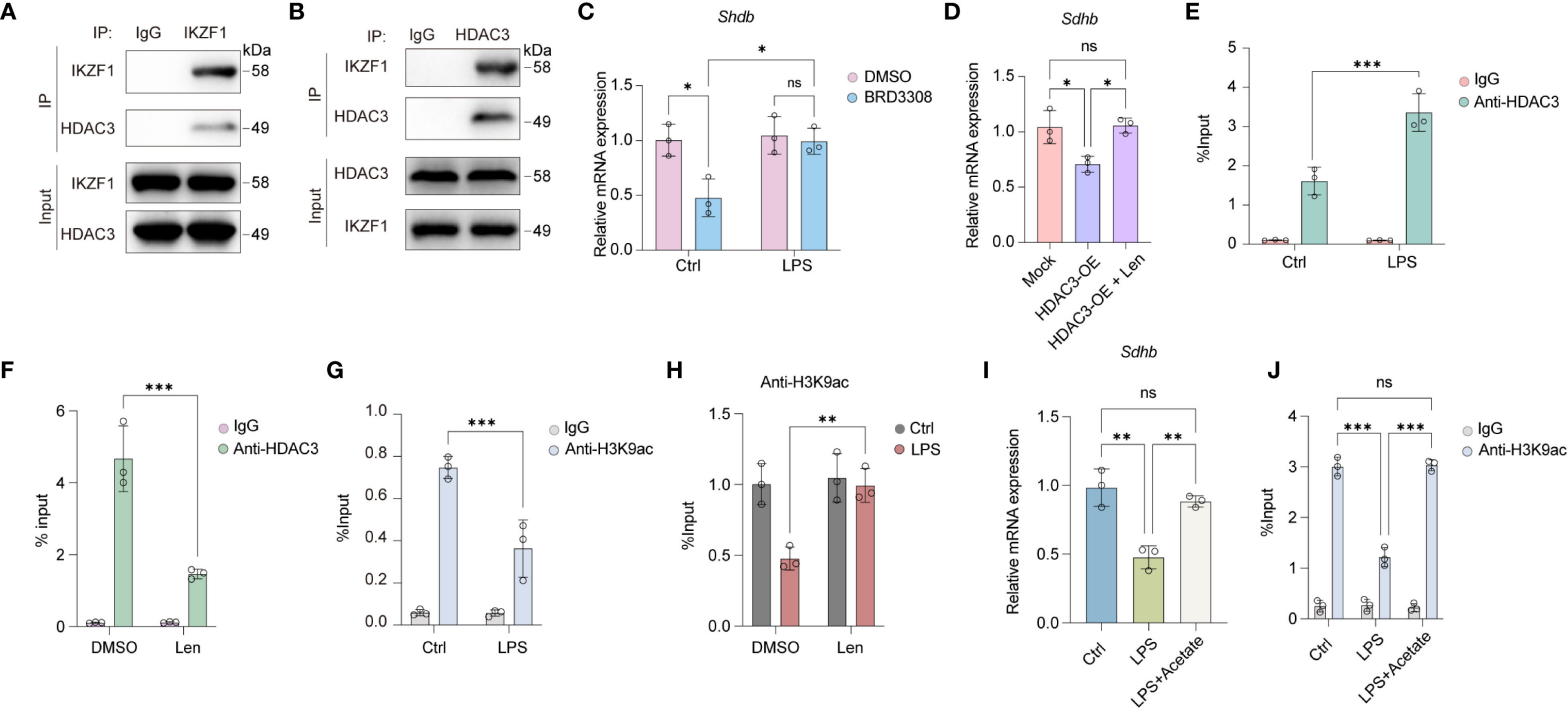
IKZF1 interacts with HDAC3 to regulate SHDB expression. **(A)** IP analysis of the interaction between IKZF1 and HDAC3 under LPS stimulation. **(B)** IP analysis of the interaction between IKZF1 and HDAC3 under LPS stimulation. **(C)** RT-qPCR analysis of *Shdb* mRNA expression in peritoneal macrophages treated with HDAC3 inhibitor BRD3308 or left untreated with LPS treatment or not. (n=3 per group). **(D)** RT-qPCR analysis of *Shdb* mRNA expression in peritoneal macrophages transfected with HDAC3 overexpression plasmid or mock, followed by Len treatment. (n=3 per group). **(E)** ChIP-qPCR analysis of HDAC3 enrichment at *Shdb* promoter in peritoneal macrophages treated with LPS or left untreated (Ctrl). (n=3 per group). **(F)** ChIP-qPCR analysis of HDAC3 enrichment at *Shdb* promoter in peritoneal macrophages treated with Len or left untreated. (n=3 per group). **(G)** ChIP-qPCR analysis of H3K9ac enrichment at *Shdb* promoter in peritoneal macrophages treated with LPS or left untreated (Ctrl). (n=3 per group). **(H)** ChIP-qPCR analysis of H3K9ac enrichment at *Shdb* promoter in peritoneal macrophages treated with Len or left untreated followed with LPS treatment or left untreated (Ctrl). (n=3 per group). **(I)** RT-qPCR analysis of *Shdb* mRNA expression treated with LPS or left untreated (Ctrl), followed by acetate treatment. (n=3 per group). **(J)** ChIP-qPCR analysis of H3K9ac enrichment at *Shdb* promoter in peritoneal macrophages treated with LPS or left untreated (Ctrl) followed by acetate treatment. (n=3 per group). All data are presented as mean ± SD. One-way ANOVA **(D, I)**; Two-way ANOVA **(C, E–H, J)**. *p < 0.05, **p < 0.01, ***p < 0.001; ns, non- significant.

We further explored the mechanism by which IKZF1 suppresses the expression of SDHB. Previous studies have demonstrated that transcription factors can mediate transcriptional repression of downstream genes by recruiting histone deacetylases. Through co-immunoprecipitation experiments, we confirmed that IKZF1 can directly bind to HDAC3 (**Figures 6A, B**). Following LPS stimulation, there was a notable decrease in *Sdhb* expression within peritoneal macrophages, which was significantly counteracted by the inhibition of HDAC3 with BRD3308 (**Figure 6C**), suggesting that the suppression of *Sdhb* expression might be achieved through the recruitment of HDAC3 by IKZF1. Our results revealed that the forced expression of HDAC3 significantly dampened *Sdhb* expression, and this inhibitory effect was mitigated by the inhibition of IKZF1 (**Figure 6D**), indicating that HDAC3 functions in an IKZF1-dependent manner. Further ChIP-qPCR analysis revealed that LPS induced the enrichment of HDAC3 in the Sdhb promoter region, which decreased after inhibiting IKZF1 (**Figures 6E, F**). Subsequently, we performed ChIP-qPCR analysis with H3K9ac antibody and found that the H3K9ac modification in the *Sdhb* promoter region was significantly reduced after LPS stimulation (**Figure 6G**). Inhibition of IKZF1 with Len significantly improves H3K9ac modification in the *Sdhb* promoter region (**Figure 6H**). Acetate serves as a critical precursor for the synthesis of acetyl-CoA, which is a key molecule in the process of histone acetylation. By supplementing with acetate, the cellular levels of acetyl-CoA can be elevated, thereby enhancing the acetylation of histones. We found that acetate supplementation significantly reversed the LPS-induced reduction in *Sdhb* expression (**Figure 6I**). Moreover, the reduction in H3K9ac modification of *Sdhb* induced by LPS was also restored (**Figure 6J**). Overall, IKZF1 collaborates with HDAC3 to inhibit *Sdhb* expression, which in turn exacerbates macrophage inflammatory responses triggered by peritonitis.

## Discussion

IKZF1 plays a significant regulatory role in myeloid cells, which are crucial components of the innate immune system (19, 21, 23). In the context of myeloid cells, IKZF1 has been implicated in several key functions. IKZF1 can act as both a transcriptional activator and repressor, modulating the expression of target genes in myeloid cells (22). This dual role allows IKZF1 to fine-tune the inflammatory response and other immune functions (23). IKZF1 is involved in chromatin remodeling, which affects the accessibility of DNA to transcription factors and thus influences gene expression (29). This function is particularly relevant in the context of inflammatory responses, where rapid changes in gene expression are required. The multifaceted role of IKZF1 in myeloid cells underscores its importance in immune regulation and highlights it as a potential therapeutic target for modulating inflammatory diseases and other conditions involving the innate immune system (30). Here we reveal an important regulatory role for IKZF1 in the progression of acute peritonitis. We demonstrated that IKZF1 is an important driver of the macrophage inflammatory response. IKZF1 inhibits SDHB expression through epigenetic mechanisms, leading to macrophage mitochondrial dysfunction. Our findings provide a new perspective and theoretical basis for the treatment of acute peritonitis.

Macrophages, which are central to inflammatory responses, show high plasticity and adopt different activation states according to the stimulus signals from the tissue microenvironment, switching between pro-inflammatory and anti-inflammatory states (17). Recent studies have highlighted the importance of metabolic reprogramming in macrophages, which involves various cytoplasmic and mitochondrial pathways such as glycolysis, oxidative phosphorylation, and the pentose phosphate pathway, to meet the high energy demands during immune responses (31). During acute inflammation, pro-inflammatory macrophages enhance glycolysis and lipid synthesis for energy production, while during resolution, they shift to a more oxidative state with intact TCA cycle and high oxidative phosphorylation for clearance of debris (32). Understanding the interplay between mitochondrial function and macrophage inflammation is essential for developing therapeutic strategies to modulate inflammation and promote resolution.

Mitochondria is also crucial for the activation, differentiation, and survival of macrophages and other immune cells (33). Changes in mitochondrial physiology in response to various extracellular signals can underlie the state of macrophage activation (34, 35). These alterations encompass changes in oxidative metabolism, mitochondrial membrane potential, tricarboxylic acid (TCA) cycle activity, the release of mitochondrial reactive oxygen species (ROS) and mitochondrial DNA, as well as transformations in mitochondrial ultrastructure (36–38). The proximity of mtDNA to the site of ROS production and its lack of protective histones make it particularly susceptible to mutations, leading to further genomic instability and respiratory chain dysfunction (39). This mechanism can regulate innate immunity by influencing redox-sensitive inflammatory pathways and directly activating the inflammasome, thereby causing excessive activation of the inflammatory response (38, 40, 41). In disease settings, mitochondrial dysfunction and oxidative stress contribute to the dysregulation of the inflammatory response, highlighting the importance of mitochondria as a dynamic signal source that regulates macrophage biology to fine-tune immune responses (42). Researchers are exploring various approaches to maintain mitochondrial function, including the application of antioxidant compounds, the creation of therapies that promote mitophagy, and the pharmacological modulation of mitochondrial sirtuins, to reduce ROS production and curb inflammation (43). Effective strategies are needed to enhance our comprehension of how mitochondrial dysfunction contributes to the inflammatory aspects of various acute diseases. Gaining this knowledge is crucial for devising new therapies that can enhance human health. Utilizing unmanipulated cells from both healthy and diseased donors can help prevent conflicting results and offer a clearer picture of mitochondrial function in inflammation. Understanding the molecular mechanisms underlying these interactions is crucial for developing targeted therapies to mitigate inflammation and improve outcomes in inflammatory conditions. Our study demonstrated that IKZF1 inhibition significantly enhanced macrophage oxidative phosphorylation as well as inhibited the accumulation of glycolytic lactate production, confirming that IKZF1 enhanced the pro-inflammatory glycolytic metabolic shift in macrophages. Targeting macrophage metabolic patterns by IKZF1 may provide directional insights for the prevention of multiple inflammatory diseases.

SDHB deficiency triggers inflammation through a complex interplay of metabolic and signaling disruptions within macrophages (44). As a crucial component of the mitochondrial electron transport chain and the tricarboxylic acid (TCA) cycle, SDHB is essential for cellular respiration and energy metabolism (45, 46). When SDHB is deficient, the normal flow of metabolites through the TCA cycle is impeded, leading to the accumulation of succinate (47). Accumulated succinate acts as a competitive inhibitor of α-ketoglutarate-dependent dioxygenases, which are involved in the degradation of hypoxia-inducible factors (HIFs) (48). This inhibition stabilizes HIFs, leading to the activation of genes associated with inflammation and cell survival (49, 50). The same enzymatic inhibition also affects the demethylation of histones, DNA, and RNA, leading to epigenetic changes that can alter gene expression patterns and promote a pro-inflammatory state (51, 52). Furthermore, SDHB deficiency results in the excessive production of mitochondrial reactive oxygen species, which can further activate inflammatory pathways and inhibit crucial signaling molecules such as Stat3 (53). The inhibition of Stat3 tyrosine phosphorylation is a key event in the suppression of IL-10 production, an anti-inflammatory cytokine that acts as a negative feedback regulator in macrophages (54). In summary, SDHB deficiency leads to a metabolic and epigenetic reprogramming in macrophages that promotes inflammation, in part by disrupting the balance between pro-inflammatory and anti-inflammatory signals. This understanding of the mechanisms provides insights into the pathophysiology of diseases associated with SDHB mutations and may guide the development of targeted therapies to modulate inflammation. Based on our research findings, we discovered that inhibiting IKZF1 significantly restores the expression of SDHB, reduces the production of succinate and ROS, thereby improving the inflammatory response in macrophages. Therefore, the alteration of IKZF1 in peritonitis and its potential therapeutic role deserve further exploration.

Histone acetylation, an epigenetic mark that involves the addition of an acetyl group to lysine residues on histone proteins, is closely associated with the activation of gene transcription (55). This modification enhances chromatin accessibility, allowing for the recruitment of transcriptional machinery and thereby influencing the expression of genes involved in inflammation (56, 57). In the context of macrophage activation, histone acetylation can lead to the upregulation of pro-inflammatory cytokines and chemokines, which are essential for mounting an effective immune response (58). However, dysregulation of histone acetylation can contribute to chronic inflammation and the pathogenesis of various diseases, including autoimmune and inflammatory disorders. Recent studies have highlighted the potential of targeting histone acetylation as a therapeutic strategy to modulate macrophage function and resolve inflammation. For instance, the use of histone deacetylase (HDAC) inhibitors has been shown to attenuate inflammatory responses in macrophages by altering the acetylation status of transcription factors such as NF-κB and STAT3, thereby dampening the production of inflammatory mediators (59). These findings underscore the importance of understanding the interplay between histone acetylation and macrophage inflammation, offering new avenues for developing targeted therapies to treat inflammatory diseases. H3K9ac, a specific histone acetylation modification, is intimately connected with the inflammatory response in macrophages (60). As a marker of active gene transcription, H3K9ac plays a crucial role in modulating the expression of genes involved in inflammation. In the context of macrophage activation, H3K9ac can enhance the transcription of pro-inflammatory cytokines and chemokines, which are vital for the immune response (61). Previous studies have shown that the level of H3K9ac can change in response to inflammatory stimuli, such as LPS, leading to alterations in chromatin structure and gene expression patterns that promote a pro-inflammatory phenotype in macrophages. In this study, we discovered that IKZF1 collaborates with HDAC3 to regulate the H3K9ac modification, thereby affecting the functional transition of macrophages and participating in the pathological process of acute peritonitis.

Beyond the macrophage-centric findings of the present study, several independent lines of evidence have established non-redundant roles for IKZF1 in defined cellular compartments. In regulatory T cells, chromatin immunoprecipitation followed by deep sequencing has demonstrated that IKZF1 co-occupies the FOXP3-bound enhancer at the *Il2* locus, leading to transcriptional repression and maintenance of Treg stability (22). In the natural killer cell lineage, germline deletion of Ikzf1 arrests development at an immature CD27^+^CD11b^-^ stage and abolishes granzyme-B–mediated cytotoxicity against MHC-I-deficient targets (30). Within B lymphopoiesis, both patient-derived sequencing and murine transplantation studies reveal that IKZF1 directly transactivates PAX5 and EBF1 while repressing BCL6, a transcriptional circuit required for the pro-B to pre-B transition and recurrently disrupted in B-cell acute lymphoblastic leukaemia (19, 62, 63). These findings collectively delineate the cell-type-specific regulatory landscape mediated by IKZF1, highlighting the necessity for context-dependent therapeutic approaches when targeting this transcription factor in pathological conditions beyond acute peritonitis.

The study presents compelling insights into the role of IKZF1 in exacerbating macrophage-driven inflammation during acute peritonitis through epigenetic modulation of mitochondrial function. However, several limitations warrant consideration. First, the reliance on a murine CLP model, while widely accepted for studying peritonitis, introduces challenges in translating findings to human pathophysiology. Species-specific differences in immune responses, mitochondrial biology, and disease progression may limit the direct applicability of the results to clinical settings. For instance, human macrophages or patient-derived samples were not analyzed, which could have provided critical validation of the proposed IKZF1/HDAC3-SDHB axis in human disease. Clinically, while Lenalidomide’s therapeutic potential is highlighted, the study does not address long-term safety concerns of IKZF1 inhibition, such as immune suppression or off-target toxicity, which are critical for translational applications. Addressing these limitations in future studies could refine the mechanistic understanding of IKZF1 in inflammation and improve the translational relevance of targeting this pathway in peritonitis.

## Conclusion

Collectively, our results validate that the decrease in *Sdhb* expression triggered by LPS can be attributed to the upregulated expression of IKZF1. Decreased Sdhb led to mitochondrial dysfunction, enhanced accumulation of proinflammatory mediators, and ultimately exacerbated macrophage inflammatory responses. Mechanistically, IKZF1 recruits HDAC3, which then mediates the removal of H3K9ac modification. This process ultimately suppresses *Sdhb* expression and facilitates inflammatory responses within macrophages. Therefore, IKZF1 is expected to be an efficient pharmaceutical ingredient, providing new strategies for treating peritonitis.

## Data Availability

The original contributions presented in the study are included in the article/**Supplementary Material**. Further inquiries can be directed to the corresponding author/s.

## References

[1] Zheng M Wu X Xu Y Ma S Shen J Li T. Curcumin reverses high-level tigecycline resistance mediated by different mechanisms in Gram-negative bacteria. Phytomedicine. (2024) 136:156319. doi: 10.1016/j.phymed.2024.156319, PMID: 39724849

[2] Durazo-Martinez K Chaudhari J Kumari S Vu HLX. Porcine peritoneal macrophages are susceptible to porcine reproductive and respiratory syndrome virus infection. Front Microbiol. (2024) 15:1505900. doi: 10.3389/fmicb.2024.1505900, PMID: 39723134 PMC11668767

[3] Maul LV Jamiolkowski D Lapides RA Mueller AM Hauschild A Garbe C. Health economic consequences associated with COVID-19-related delay in melanoma diagnosis in Europe. JAMA Netw Open. (2024) 7:e2356479. doi: 10.1001/jamanetworkopen.2023.56479, PMID: 38363565 PMC10873772

[4] Khan S Linganna M. Diagnosis and management of ascites, spontaneous bacterial peritonitis, and hepatorenal syndrome. Cleve Clin J Med. (2023) 90:209–13. doi: 10.3949/ccjm.90a.22028, PMID: 37011958

[5] Li J Xiao F Lin B Huang Z Wu M Ma H. Ferrostatin-1 improves acute sepsis-induced cardiomyopathy via inhibiting neutrophil infiltration through impaired chemokine axis. Front Cell Dev Biol. (2024) 12:1510232. doi: 10.3389/fcell.2024.1510232, PMID: 39726718 PMC11669711

[6] Qu S Yang S Xu Q Zhang M Gao F Wu Y. A milk extracellular vesicle-based nanoplatform enhances combination therapy against multidrug-resistant bacterial infections. Adv Sci (Weinh). (2024):e2406496. doi: 10.1002/advs.202406496, PMID: 39721033 PMC11831456

[7] Zhou Y Yuan Y Yao X Wang L Yao L Tang D. miPEP31 alleviates sepsis development by regulating Chi3l1-dependent macrophage polarization. Biol Direct. (2024) 19:117. doi: 10.1186/s13062-024-00568-w, PMID: 39558383 PMC11575066

[8] Wada T Senokuchi T Shi Y Furusho T Morita Y Sarie M. Orally administrated acetate inhibits atherosclerosis progression through AMPK activation via GPR43 in plaque macrophages. Atherosclerosis. (2024) 401:119088. doi: 10.1016/j.atherosclerosis.2024.119088, PMID: 39705906

[9] HuYXYouHMBaiMRYueWHLiFFHuBW. Macrophage P2Y12 regulates iron transport and its inhibition protects against atherosclerosis. J Adv Res. (2024) S2090-1232(24)00597-6. doi: 10.1016/j.jare.2024.12.019, PMID: 39674499

[10] Xu Y Xu L Zhang T Tian H Lu Y Jiang S. Antimicrobial Peptide CATH-2 Attenuates Avian Pathogenic E. coli-Induced Inflammatory Response via NF-kappaB/NLRP3/MAPK Pathway and Lysosomal Dysfunction in Macrophages. Int J Mol Sci. (2024) 25:12572. doi: 10.3390/ijms252312572, PMID: 39684284 PMC11641483

[11] Ren Y Liu Y Pang R Xu G Lei Y Kwok HF. ZIKV prM hijacks PIM1 kinase for phosphorylation to prevent ubiquitin-mediated degradation and facilitate viral replication. Front Cell Infect Microbiol. (2024) 14:1502770. doi: 10.3389/fcimb.2024.1502770, PMID: 39679197 PMC11638163

[12] Gross JL Basu R Bradfield CJ Sun J John SP Das S. Bactericidal antibiotic treatment induces damaging inflammation via TLR9 sensing of bacterial DNA. Nat Commun. (2024) 15:10359. doi: 10.1038/s41467-024-54497-3, PMID: 39609397 PMC11605096

[13] Wang W Ren Y Yu Q Jiang L Yu C Yue Z. Biodegradable exosome-engineered hydrogels for the prevention of peritoneal adhesions via anti-oxidation and anti-inflammation. Mater Today Bio. (2024) 29:101312. doi: 10.1016/j.mtbio.2024.101312, PMID: 39525394 PMC11550211

[14] Kruger AJ. Can macrophages in cirrhotic ascites fluid predict clinical outcome in spontaneous bacterial peritonitis? Gastroenterology. (2020) 158:1540–3. doi: 10.1053/j.gastro.2020.02.040, PMID: 32135164 PMC7292615

[15] Sach M Bauermeister K Burger JA Loetscher P Elsner J Schollmeyer P. Inverse MCP-1/IL-8 ratio in effluents of CAPD patients with peritonitis and in isolated cultured human peritoneal macrophages. Nephrol Dial Transpl. (1997) 12:315–20. doi: 10.1093/ndt/12.2.315, PMID: 9132652

[16] Bone-Larson CL Hogaboam CM Steinhauser ML Oliveira SH Lukacs NW Strieter RM. Novel protective effects of stem cell factor in a murine model of acute septic peritonitis. Dependence on MCP-1. Am J Pathol. (2000) 157:1177–86. doi: 10.1016/S0002-9440(10)64633-0, PMID: 11021822 PMC1850153

[17] Wynn TA Vannella KM. Macrophages in tissue repair, regeneration, and fibrosis. Immunity. (2016) 44:450–62. doi: 10.1016/j.immuni.2016.02.015, PMID: 26982353 PMC4794754

[18] Lazarov T Juarez-Carreno S Cox N Geissmann F. Publisher Correction: Physiology and diseases of tissue-resident macrophages. Nature. (2023) 619:E51. doi: 10.1038/s41586-023-06386-w, PMID: 37400553

[19] Stanulla M Cave H Moorman AV. IKZF1 deletions in pediatric acute lymphoblastic leukemia: still a poor prognostic marker? Blood. (2020) 135:252–60. doi: 10.1182/blood.2019000813, PMID: 31821407 PMC7019035

[20] Yagi T Hibi S Takanashi M Kano G Tabata Y Imamura T. High frequency of Ikaros isoform 6 expression in acute myelomonocytic and monocytic leukemias: implications for up-regulation of the antiapoptotic protein Bcl-XL in leukemogenesis. Blood. (2002) 99:1350–5. doi: 10.1182/blood.V99.4.1350, PMID: 11830486

[21] Kadono K Kageyama S Nakamura K Hirao H Ito T Kojima H. Myeloid Ikaros-SIRT1 signaling axis regulates hepatic inflammation and pyroptosis in ischemia-stressed mouse and human liver. J Hepatol. (2022) 76:896–909. doi: 10.1016/j.jhep.2021.11.026, PMID: 34871625 PMC9704689

[22] Ichiyama K Long J Kobayashi Y Horita Y Kinoshita T Nakamura Y. Transcription factor Ikzf1 associates with Foxp3 to repress gene expression in Treg cells and limit autoimmunity and anti-tumor immunity. Immunity. (2024) 57:2043–60.e10. doi: 10.1016/j.immuni.2024.07.010, PMID: 39111316

[23] Hoshino A Picard BH Polychronopoulou S Kelaidi C Azarnoush S Kracker S. Loss-of-phosphorylation of IKZF1 results in gain-of-function associated with immune dysregulation. J Allergy Clin Immunol. (2024) 154:229–36.e2. doi: 10.1016/j.jaci.2024.01.029, PMID: 38438084

[24] Hwang JS Kim KH Park J Kim SM Cho H Lee Y. Glucosamine improves survival in a mouse model of sepsis and attenuates sepsis-induced lung injury and inflammation. J Biol Chem. (2019) 294:608–22. doi: 10.1074/jbc.RA118.004638, PMID: 30455348 PMC6333887

[25] Reynier F de Vos AF Hoogerwerf JJ Bresser P van der Zee JS Paye M. Gene expression profiles in alveolar macrophages induced by lipopolysaccharide in humans. Mol Med. (2012) 18:1303–11. doi: 10.2119/molmed.2012.00230, PMID: 22952057 PMC3521791

[26] Wang Z Guo Z Wang X Chai Y Wang Z Liao H. EZH2 contributes to sepsis-induced acute lung injury through regulating macrophage polarization. Biochim Biophys Acta Mol Basis Dis. (2025) 1871:167554. doi: 10.1016/j.bbadis.2024.167554, PMID: 39471914

[27] Hildebrand DG Alexander E Hörber S Lehle S Obermayer K Münck NA. IκBζ is a transcriptional key regulator of CCL2/MCP-1. J Immunol. (2013) 190:4812–20. doi: 10.4049/jimmunol.1300089, PMID: 23547114

[28] Fink EC Ebert BL. The novel mechanism of lenalidomide activity. Blood. (2015) 126:2366–9. doi: 10.1182/blood-2015-07-567958, PMID: 26438514 PMC4653765

[29] Kim J Sif S Jones B Jackson A Koipally J Heller E. Ikaros DNA-binding proteins direct formation of chromatin remodeling complexes in lymphocytes. Immunity. (1999) 10:345–55. doi: 10.1016/S1074-7613(00)80034-5, PMID: 10204490

[30] Goh W Sudholz H Foroutan M Scheer S Pfefferle A Delconte RB. IKAROS and AIOLOS directly regulate AP-1 transcriptional complexes and are essential for NK cell development. Nat Immunol. (2024) 25:240–55. doi: 10.1038/s41590-023-01718-4, PMID: 38182668

[31] Li M Yang Y Xiong L Jiang P Wang J Li C. Metabolism, metabolites, and macrophages in cancer. J Hematol Oncol. (2023) 16:80. doi: 10.1186/s13045-023-01478-6, PMID: 37491279 PMC10367370

[32] Mills EL O’Neill LA. Reprogramming mitochondrial metabolism in macrophages as an anti-inflammatory signal. Eur J Immunol. (2016) 46:13–21. doi: 10.1002/eji.201445427, PMID: 26643360

[33] Nassef MZ Hanke JE Hiller K. Mitochondrial metabolism in macrophages. Am J Physiol Cell Physiol. (2021) 321:C1070–C81. doi: 10.1152/ajpcell.00126.2021, PMID: 34705584 PMC8959580

[34] Lampropoulou V Sergushichev A Bambouskova M Nair S Vincent EE Loginicheva E. Itaconate links inhibition of succinate dehydrogenase with macrophage metabolic remodeling and regulation of inflammation. Cell Metab. (2016) 24:158–66. doi: 10.1016/j.cmet.2016.06.004, PMID: 27374498 PMC5108454

[35] Zeng W Li F Jin S Ho PC Liu PS Xie X. Functional polarization of tumor-associated macrophages dictated by metabolic reprogramming. J Exp Clin Cancer Res. (2023) 42:245. doi: 10.1186/s13046-023-02832-9, PMID: 37740232 PMC10517486

[36] Georgakopoulos ND Wells G Campanella M. The pharmacological regulation of cellular mitophagy. Nat Chem Biol. (2017) 13:136–46. doi: 10.1038/nchembio.2287, PMID: 28103219

[37] Arnold PK Jackson BT Paras KI Brunner JS Hart ML Newsom OJ. A non-canonical tricarboxylic acid cycle underlies cellular identity. Nature. (2022) 603:477–81. doi: 10.1038/s41586-022-04475-w, PMID: 35264789 PMC8934290

[38] Hu MM Shu HB. Mitochondrial DNA-triggered innate immune response: mechanisms and diseases. Cell Mol Immunol. (2023) 20:1403–12. doi: 10.1038/s41423-023-01086-x, PMID: 37932533 PMC10687031

[39] Willems PH Rossignol R Dieteren CE Murphy MP Koopman WJ. Redox homeostasis and mitochondrial dynamics. Cell Metab. (2015) 22:207–18. doi: 10.1016/j.cmet.2015.06.006, PMID: 26166745

[40] West AP Khoury-Hanold W Staron M Tal MC Pineda CM Lang SM. Mitochondrial DNA stress primes the antiviral innate immune response. Nature. (2015) 520:553–7. doi: 10.1038/nature14156, PMID: 25642965 PMC4409480

[41] Prochnicki T Vasconcelos MB Robinson KS Mangan MSJ De Graaf D Shkarina K. Mitochondrial damage activates the NLRP10 inflammasome. Nat Immunol. (2023) 24:595–603. doi: 10.1038/s41590-023-01451-y, PMID: 36941400

[42] Wang Y Li N Zhang X Horng T. Mitochondrial metabolism regulates macrophage biology. J Biol Chem. (2021) 297:100904. doi: 10.1016/j.jbc.2021.100904, PMID: 34157289 PMC8294576

[43] Russell OM Gorman GS Lightowlers RN Turnbull DM. Mitochondrial diseases: hope for the future. Cell. (2020) 181:168–88. doi: 10.1016/j.cell.2020.02.051, PMID: 32220313

[44] Chen Z Han Z Nan H Fan J Zhan J Zhang Y. A novel pyroptosis-related gene signature for predicting the prognosis and the associated immune infiltration in colon adenocarcinoma. Front Oncol. (2022) 12:904464. doi: 10.3389/fonc.2022.904464, PMID: 35912258 PMC9330598

[45] Goncalves J Moog S Morin A Gentric G Muller S Morrell AP. Loss of SDHB promotes dysregulated iron homeostasis, oxidative stress, and sensitivity to ascorbate. Cancer Res. (2021) 81:3480–94. doi: 10.1158/0008-5472.CAN-20-2936, PMID: 34127497 PMC7616967

[46] Baysal BE Ferrell RE Willett-Brozick JE Lawrence EC Myssiorek D Bosch A. Mutations in SDHD, a mitochondrial complex II gene, in hereditary paraganglioma. Science. (2000) 287:848–51. doi: 10.1126/science.287.5454.848, PMID: 10657297

[47] Wang XH Xu S Zhou XY Zhao R Lin Y Cao J. Low chorionic villous succinate accumulation associates with recurrent spontaneous abortion risk. Nat Commun. (2021) 12:3428. doi: 10.1038/s41467-021-23827-0, PMID: 34103526 PMC8187647

[48] Baksh SC Finley LWS. Metabolic coordination of cell fate by alpha-ketoglutarate-dependent dioxygenases. Trends Cell Biol. (2021) 31:24–36. doi: 10.1016/j.tcb.2020.09.010, PMID: 33092942 PMC7748998

[49] Yuan X Ruan W Bobrow B Carmeliet P Eltzschig HK. Targeting hypoxia-inducible factors: therapeutic opportunities and challenges. Nat Rev Drug Discov. (2024) 23:175–200. doi: 10.1038/s41573-023-00848-6, PMID: 38123660 PMC12337356

[50] Bhattacharya R Brown JS Gatenby RA Ibrahim-Hashim A. A gene for all seasons: The evolutionary consequences of HIF-1 in carcinogenesis, tumor growth and metastasis. Semin Cancer Biol. (2024) 102-103:17–24. doi: 10.1016/j.semcancer.2024.06.003, PMID: 38969311

[51] Lopez-Moyado IF Ko M Hogan PG Rao A. TET enzymes in the immune system: from DNA demethylation to immunotherapy, inflammation, and cancer. Annu Rev Immunol. (2024) 42:455–88. doi: 10.1146/annurev-immunol-080223-044610, PMID: 38360546

[52] Shen L Song CX He C Zhang Y. Mechanism and function of oxidative reversal of DNA and RNA methylation. Annu Rev Biochem. (2014) 83:585–614. doi: 10.1146/annurev-biochem-060713-035513, PMID: 24905787 PMC4786441

[53] Gobelli D Serrano-Lorenzo P Esteban-Amo MJ Serna J Perez-Garcia MT Orduna A. The mitochondrial succinate dehydrogenase complex controls the STAT3-IL-10 pathway in inflammatory macrophages. iScience. (2023) 26:107473. doi: 10.1016/j.isci.2023.107473, PMID: 37575201 PMC10416071

[54] Zhang HF Wu MX Lin YQ Xie SL Huang TC Liu PM. IL-33 promotes IL-10 production in macrophages: a role for IL-33 in macrophage foam cell formation. Exp Mol Med. (2017) 49:e388. doi: 10.1038/emm.2017.183, PMID: 29099095 PMC5704190

[55] An YJ Jo S Kim JM Kim HS Kim HY Jeon SM. Lactate as a major epigenetic carbon source for histone acetylation via nuclear LDH metabolism. Exp Mol Med. (2023) 55:2238–47. doi: 10.1038/s12276-023-01095-w, PMID: 37779146 PMC10618192

[56] Wu CJ Xu X Yuan DY Liu ZZ Tan LM Su YN. Arabidopsis histone acetyltransferase complex coordinates cytoplasmic histone acetylation and nuclear chromatin accessibility. Sci Adv. (2024) 10:eadp1840. doi: 10.1126/sciadv.adp1840, PMID: 39630902 PMC11616720

[57] De Sá Fernandes C Novoszel P Gastaldi T Krauß D Lang M Rica R. The histone deacetylase HDAC1 controls dendritic cell development and anti-tumor immunity. Cell Rep. (2024) 43:114308. doi: 10.1016/j.celrep.2024.114308, PMID: 38829740

[58] Jiao YX Zhou YM Zhou ZW He Y Liu S Xu XT. Histone acetylation alteration by KAT6A inhibitor WM-1119 suppresses IgE-mediated mast cell activation and allergic inflammation via reduction in AP-1 signaling. Biochem Pharmacol. (2024) 232:116732. doi: 10.1016/j.bcp.2024.116732, PMID: 39709039

[59] Marques O Horvat NK Zechner L Colucci S Sparla R Zimmermann S. Inflammation-driven NFκB signaling represses Ferroportin transcription in macrophages via HDAC 1 and 3. Blood. (2024) 145:866–80. doi: 10.1182/blood-2024-210131 39656097

[60] Li J Ye F Xu X Xu P Wang P Zheng G. Targeting macrophage M1 polarization suppression through PCAF inhibition alleviates autoimmune arthritis via synergistic NF-κB and H3K9Ac blockade. J Nanobiotechnol. (2023) 21:280. doi: 10.1186/s12951-023-02012-z, PMID: 37598147 PMC10439630

[61] Sun C An Q Li R Chen S Gu X An S. Calcitonin gene-related peptide induces the histone H3 lysine 9 acetylation in astrocytes associated with neuroinflammation in rats with neuropathic pain. CNS Neurosci Ther. (2021) 27:1409–24. doi: 10.1111/cns.13720, PMID: 34397151 PMC8504526

[62] Heltemes-Harris LM Hubbard GK LaRue RS Munro SA Yang R Henzler CM. Identification of mutations that cooperate with defects in B cell transcription factors to initiate leukemia. Oncogene. (2021) 40:6166–79. doi: 10.1038/s41388-021-02012-z, PMID: 34535769 PMC8556320

[63] Vairy S Tran TH. IKZF1 alterations in acute lymphoblastic leukemia: The good, the bad and the ugly. Blood Rev. (2020) 44:100677. doi: 10.1016/j.blre.2020.100677, PMID: 32245541

