# Supplementary material for: IKZF1 exacerbates the inflammatory response by epigenetically modulating mitochondrial function following acute peritonitis
Source: Front Immunol. 2025 Sep 12;16:1600903. doi: 10.3389/fimmu.2025.1600903 (PMC12463629; doi:10.3389/fimmu.2025.1600903)
Supplement: Supplementary file 1 [file Table1.docx]

**Supplementary Material**

**IKZF1 exacerbates the inflammatory response by epigenetically modulating mitochondrial function following acute peritonitis**

Guanya Liu, Pengfei Hu, Ying Dong, Yamin Xu, Zhengyao Yang, Zihao Qi, Yuantao Su

Table S1: qRT-PCR primers used for each target gene

| Primers | Sequence (5’-3’) |
| --- | --- |
| *Ikzf1*-Forward | ATGTCCCAAGTTTCAGGAAAGG |
| *Ikzf1*-Reverse | GCACGCCCATTCTCTTCATC |
| *Il1b*-Forward | GCAACTGTTCCTGAACTCAACT |
| *Il1b*-Reverse | ATCTTTTGGGGTCCGTCAACT |
| *Il6*-Forward | TAGTCCTTCCTACCCCAATTTCC |
| *Il6*-Reverse | TTGGTCCTTAGCCACTCCTTC |
| *Tnfa*-Forward | CCCTCACACTCAGATCATCTTCT |
| *Tnfa*-Reverse | GCTACGACGTGGGCTACAG |
| *Sdha*-Forward | GGAACACTCCAAAAACAGACCT |
| *Sdha*-Reverse | CCACCACTGGGTATTGAGTAGAA |
| *Sdhb*-Forward | AATTTGCCATTTACCGATGGGA |
| *Sdhb*-Reverse | AGCATCCAACACCATAGGTCC |
| *Sdhc*-Forward | GCTGCGTTCTTGCTGAGACA |
| *Sdhc*-Reverse | ATCTCCTCCTTAGCTGTGGTT |
| *Sdhd*-Forward | TGGTCAGACCCGCTTATGTG |
| *Sdhd*-Reverse | GGTCCAGTGGAGAGATGCAG |
| *Gapdh-*Forward | AGGTCGGTGTGAACGGATTTG |
| *Gapdh-*Reverse | TGTAGACCATGTAGTTGAGGTCA |
| *Hif1a-*Forward | ACCTTCATCGGAAACTCCAAAG |
| *Hif1a-*Reverse | CTGTTAGGCTGGGAAAAGTTAGG |
| *Il12b-*Forward | TGGTTTGCCATCGTTTTGCTG |
| *Il12b-*Reverse | ACAGGTGAGGTTCACTGTTTCT |
| *Il10-*Forward | GCTCTTACTGACTGGCATGAG |
| *Il10-*Reverse | CGCAGCTCTAGGAGCATGTG |
| *Arg1-*Forward | CTCCAAGCCAAAGTCCTTAGAG |
| *Arg1-*Reverse | AGGAGCTGTCATTAGGGACATC |
| *Mrc1-*Forward | CTCTGTTCAGCTATTGGACGC |
| *Mrc1-*Reverse | CGGAATTTCTGGGATTCAGCTTC |
| *Col1a1-*Forward | GCTCCTCTTAGGGGCCACT |
| *Col1a1-*Reverse | CCACGTCTCACCATTGGGG |
| *Col3a1-*Forward | CTGTAACATGGAAACTGGGGAAA |
| *Col3a1-*Reverse | CCATAGCTGAACTGAAAACCACC |
| *Acta2-*Forward | GTCCCAGACATCAGGGAGTAA |
| *Acta2-*Reverse | TCGGATACTTCAGCGTCAGGA |
| *Mff-*Forward | ATGCCAGTGTGATAATGCAAGT |
| *Mff-*Reverse | CTCGGCTCTCTTCGCTTTG |
| *Fis1-*Forward | TGTCCAAGAGCACGCAATTTG |
| *Fis1-*Reverse | CCTCGCACATACTTTAGAGCCTT |
| *Mfn1-*Forward | CCTACTGCTCCTTCTAACCCA |
| *Mfn1-*Reverse | AGGGACGCCAATCCTGTGA |
| *Mfn2-*Forward | TGACCTGAATTGTGACAAGCTG |
| *Mfn2-*Reverse | AGACTGACTGCCGTATCTGGT |

Table S2: Primer sequences for ChIP

| Primers | Sequence (5’-3’) |
| --- | --- |
| *Sdhb*-Forward | TTACCACAGCATTCCAGAG |
| *Sdhb*-Forward | GGAGACAGTAGGAGAACCA |
