# Supplementary material for: IKZF1 exacerbates the inflammatory response by epigenetically modulating mitochondrial function following acute peritonitis
Source: Front Immunol. 2025 Sep 12;16:1600903. doi: 10.3389/fimmu.2025.1600903 (PMC12463629; doi:10.3389/fimmu.2025.1600903)
Supplement: Supplementary file 2 [file DataSheet1.docx]

**Supplementary Material**

**IKZF1 exacerbates the inflammatory response by epigenetically modulating mitochondrial function following acute peritonitis**

Guanya Liu, Pengfei Hu, Ying Dong, Yamin Xu, Zhengyao Yang, Zihao Qi, Yuantao Su

**Supplementary Figures**

^
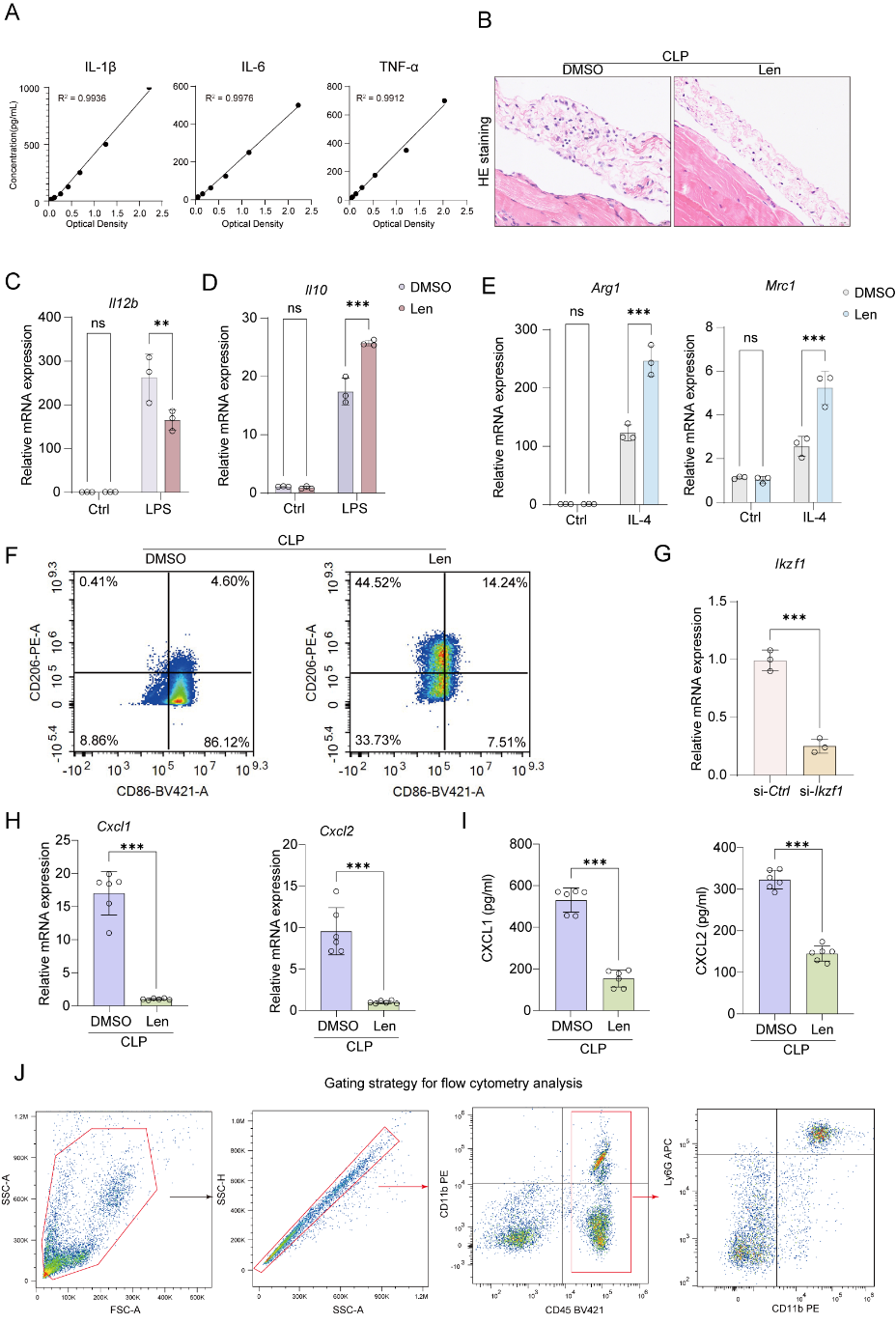
^

**Supplemental Figure 1. IKZF1 exacerbates the inflammatory response of macrophages.** (A) Standard Curves for the detection of IL-1β, IL-6, and TNF-α. (B) H&E staining of peritoneal tissue histological sections from mice treated with the IKZF1 inhibitor Len or DMSO after CLP-induced injury. (C and D) Q-PCR analysis of *Il12b* and *Il10* in peritoneal macrophages treated with IKZF1 inhibitor Len or DMSO following LPS (100 ng/ml) stimulation for 4 h or left untreated (Ctrl). (n=3 per group). (E) Q-PCR analysis of *Arg1* and *Mrc1* in peritoneal macrophages treated with IKZF1 inhibitor Len or DMSO following IL-4 (20 ng/ml) stimulation for 12 h or left untreated (Ctrl). (n=3 per group). (F) Flow cytometry analysis of CD206 expression in peritoneal macrophages from CLP-treated mice. (G) Q-PCR analysis of *Ikzf1* in peritoneal macrophages treated with *Ikzf1* siRNA (20 nmol) or Ctrl siRNA for 48h. (n=3 per group). (H) Q-PCR analysis of *Cxcl1* and *Cxcl2* in peritoneal macrophages from mice treated with the IKZF1 inhibitor Len or DMSO after CLP-induced injury.(n=6 per group). (I) ELISA analysis of CXCL1 and CXCL2 in the peritoneal lavage fluid from mice treated with the IKZF1 inhibitor Len or DMSO after CLP-induced injury.(n=6 per group). (J) Gating strategy for flow cytometry of monocytes and neutrophils after CLP-induced injury. All data are represented as mean ± SD. Student’s t-test (G, H, I); Two-way ANOVA (C, D, E). ***p* < 0.01; ****p* < 0.001.

^
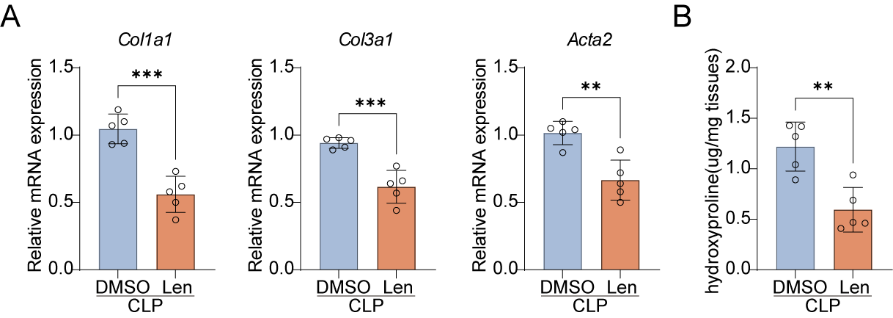
^

**Supplemental Figure 2. IKZF1 inhibition improves CLP-induced pulmonary fibrosis.** (A) Q-PCR analysis of *Col1a1*, *Col3a1,* and *Acta2* in lung tissues from mice treated with the IKZF1 inhibitor Len or DMSO after CLP-induced injury. (n=5 per group). (B) Hydroxyproline analysis of lung tissues from mice treated with the IKZF1 inhibitor Len or DMSO after CLP-induced injury. (n=5 per group). All data are represented as mean ± SD. Student’s t-test (A, B). ***p* < 0.01; ****p* < 0.001.

^
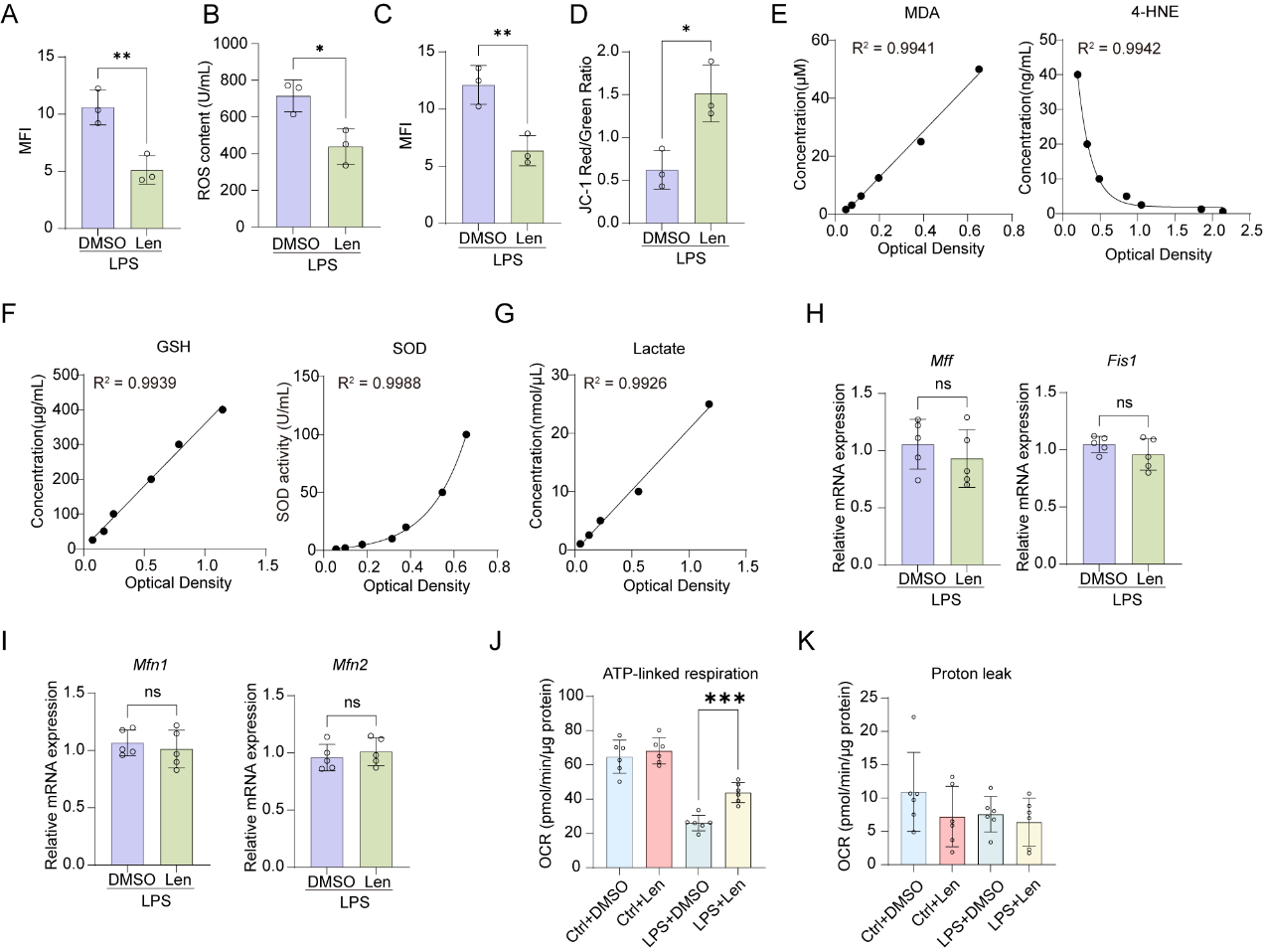
^

**Supplemental Figure 3. IKZF1 inhibition improves mitochondrial function.** (A) Quantitative results of the mean fluorescence intensity (MFI) of DHE staining. (n=3 per group). (B) ROS levels of peritoneal macrophages treated with Len or DMSO following LPS (100 ng/ml) stimulation for 4 h. (n=3 per group). (C) Quantitative results of the MFI of mitoSOX staining. (n=3 per group). (D) The ratio of the MFI of red and green fluorescence in JC-1 staining. (n=3 per group). (E-G) Standard Curves for the detection of MDA, 4-HNE, GSH, SOD, and Lactate. (H) Q-PCR analysis of *Mff* and *Fis1* in peritoneal macrophages treated with Len or DMSO following LPS (100 ng/ml) stimulation for 4 h. (n=5 per group). (I) Q-PCR analysis of *Mfn1* and *Mfn2* in peritoneal macrophages treated with Len or DMSO following LPS (100 ng/ml) stimulation for 4 h. (n=5 per group). (J and K) Relative ATP-linked respiratory and proton leak analysis of peritoneal macrophages treated with Len or DMSO following LPS (100 ng/ml) stimulation for 4 h. (n=6 per group). All data are represented as mean ± SD. Student’s t-test (A, B, C, D, H, I, J, K). **p* < 0.05; ***p* < 0.01; ****p* < 0.001.
